# BLIMP1 and NR4A3 Transcription Factors Reciprocally Regulate Antitumor CAR T-cell Stemness and Exhaustion

**DOI:** 10.1126/scitranslmed.abn7336

**Published:** 2022-11-09

**Authors:** In-Young Jung, Vivek Narayan, Sierra McDonald, Andrew J. Rech, Robert Bartoszek, Gwanui Hong, Megan M. Davis, Jun Xu, Alina C. Boesteanu, Julie S. Barber-Rotenberg, Gabriela Plesa, Simon F. Lacey, Julie K. Jadlowsky, Donald L. Siegel, Dana M. Hammill, Park F. Cho-Park, Shelley L. Berger, Naomi B. Haas, Joseph A. Fraietta

**Affiliations:** 1 Department of Microbiology, Perelman School of Medicine, University of Pennsylvania, Philadelphia, PA (19104); 2 Center for Cellular Immunotherapies, Perelman School of Medicine, University of Pennsylvania, Philadelphia, PA (19104); 3 Abramson Cancer Center, Perelman School of Medicine, University of Pennsylvania, Philadelphia, PA (19104); 4 Department of Pathology and Laboratory Medicine, Perelman School of Medicine, University of Pennsylvania, Philadelphia, PA (19104); 5 Department of Medicine, Perelman School of Medicine, University of Pennsylvania, Philadelphia, PA (19104); 6 Parker Institute for Cancer Immunotherapy, University of Pennsylvania, Philadelphia, PA (19104); 7 Epigenetics Institute, Perelman School of Medicine, University of Pennsylvania, Philadelphia, PA (19104); 8 Department of Cell and Developmental Biology, Perelman School of Medicine, University of Pennsylvania, Philadelphia, PA (19104); 9 Department of Genetics, Perelman School of Medicine, University of Pennsylvania, Philadelphia, PA (19104); 10 Department of Systems Pharmacology & Translational Therapeutics, Perelman School of Medicine, University of Pennsylvania, Philadelphia, PA (19104)

## Abstract

Chimeric antigen receptor (CAR) T cells have not induced meaningful clinical responses in solid tumors. Loss of T cell stemness, poor expansion capacity, and exhaustion during prolonged tumor antigen exposure are major causes of CAR T cell therapeutic resistance. Single-cell RNA-sequencing analysis of CAR T cells from a first-in-human trial in metastatic prostate cancer identified two independently validated cell states associated with antitumor potency or lack of efficacy. Low expression of *PRDM1*, encoding the BLIMP1 transcription factor, defined highly potent *TCF7* [encoding T cell factor 1 (TCF1)]–expressing CD8^+^ CAR T cells, whereas enrichment of *HAVCR2* [encoding T cell immunoglobulin and mucin-domain containing-3 (TIM-3)]–expressing CD8^+^ T cells with elevated *PRDM1* was associated with poor outcomes. *PRDM1* knockout promoted *TCF7*-dependent CAR T cell stemness and proliferation, resulting in marginally enhanced leukemia control in mice. However, in the setting of *PRDM1* deficiency, a negative epigenetic feedback program of nuclear factor of activated T cells (NFAT)–driven T cell dysfunction was identified. This program was characterized by compensatory up-regulation of *NR4A3* and other genes encoding exhaustion-related transcription factors that hampered T cell effector function in solid tumors. Dual knockout of *PRDM1* and *NR4A3* skewed CAR T cell phenotypes away from TIM-3^+^CD8^+^ and toward TCF1^+^CD8^+^ to counter exhaustion of tumor-infiltrating CAR T cells and improve antitumor responses, effects that were not achieved with *PRDM1* and *NR4A3* single knockout alone. These data underscore dual targeting of *PRDM1* and *NR4A3* as a promising approach to advance adoptive cell immuno-oncotherapy.

## One Sentence Summary

Two transcription factors, BLIMP1 and NR4A3, reciprocally regulate T-cell stemness and exhaustion and can be exploited for CAR T-cell therapy.

## Editor’s Summary

Tuning Up CAR T-Cells. Despite their success at treating hematological malignancies, CAR T-cells have not been as effective in treating solid tumors. This is, in part, due to loss of stemness and development of an exhausted phenotype after infusion. Here, Jung *et al.* investigated a strategy to reverse this phenotype based on analysis of CAR T-cells employed in a clinical trial for metastatic prostate cancer. The authors identified two transcription factors, BLIMP1 and NR4A3, that regulated CAR T-cell dysfunction. Dual knockout of these two transcription factors reduced CAR T-cell exhaustion and enhanced their antitumor activity in multiple murine models, outperforming single knockouts. These data suggest that transcription factor modulation may be a key strategy to improve CAR T-cells for solid tumors.

## Introduction

Chimeric antigen receptor (CAR) T-cells have induced unprecedented high rates of complete remission in relapsed and refractory B-cell malignancies. Despite these successes, a substantial portion of patients with B-cell leukemia, lymphoma, and myeloma fail to respond to CAR T-cell therapy and only small subsets of patients experience long-term durable responses (1–5). The efficacy of CAR T-cell therapy in solid tumor indications has been even more limited to date (6–8). Unlike the situation in hematologic malignancies, CAR T-cells must traffic to solid tumor sites and surmount stromal elements to infiltrate into the tumor bed and elicit antigen-directed cytotoxicity. Even if trafficking and infiltration are successful, CAR T-cells often become dysfunctional due to chronic antigen exposure and additional immunosuppressive mechanisms operative within the tumor microenvironment (TME).

CAR T-cells derived from naïve and early memory subsets have been shown to robustly expand in vivo and are long-lived with a self-renewal capacity (9–11). We and others have demonstrated that naïve or stem-like early memory T-cells genetically redirected with CARs have more durable engraftment and antitumor effector function compared to highly differentiated cells (12, 13). However, persistent tumor antigen stimulation in the setting of hematopoietic and non-hematopoietic cancers often leads to exhaustion. T-cell exhaustion is characterized by upregulation of multiple inhibitory receptors (10, 13–15), the inability to respond to homeostatic cytokines, loss of effector function (9, 16) and reduced survival (17). CAR T-cell exhaustion can also be facilitated by antigen-independent tonic signaling through the synthetic antigen receptor during ex vivo cell expansion or following infusion (18, 19).

We previously found that sustained remissions in CD19 CAR T-cell therapy of chronic lymphocytic leukemia (CLL) were associated with an increased peak expansion after infusion and relatively longer persistence. Cell products that were particularly effective showed greater proliferative capacity prior to and during treatment. Transcriptomic analysis suggested that remission correlated with early memory T-cell signatures as well as stemness (defined by the self-renewal capacity and the ability to differentiate into multiple downstream cell types), whereas gene expression profiles from non-responders were associated with terminal differentiation and exhaustion (20). Similarly, memory CD8^+^ T-cell transcriptional programs enriched in axicabtagene ciloleucel infusion products were strongly related to the long-term durability of response, whereas gene signatures of exhaustion correlated with early treatment failure in patients with large B-cell lymphomas (LBCL) (21). Importantly, it may be possible to overcome many of the above issues by using highly proliferative, “optimally programmed” CAR T-cells. However, the cellular and molecular basis for CAR T-cell-intrinsic resistance in failed therapy of solid tumors has not been elucidated. Thus, engineering approaches to generate CAR T-cells with optimal potency and other aforementioned desirable features informed by correlative analyses have been scant to date.

Here we analyzed dominant negative transforming growth factor β receptor II (TGF-βRII) ‘armored’ prostate-specific membrane antigen (PSMA) CAR T-cell infusion products from patients with metastatic castration-resistant prostate cancer (mCRPC) (clinicaltrials.gov NCT03089203) (22). Using single cell RNA-sequencing (scRNA-seq) analysis, we identified heterogeneity in the cellular and molecular features of CAR T-cells associated with variation in therapeutic product potency across multiple CAR T-cell trials. Based on these findings, we hypothesized that deletion of the PR/SET domain 1 (*PRDM1*) gene encoding the B-lymphocyte-induced maturation protein 1 (BLIMP1) transcription factor, which is upregulated in poorly functional *Hepatitis A Virus Cellular Receptor 2* (*HAVCR2*; encoding T-cell immunoglobulin and mucin-domain containing-3, TIM-3)-expressing CD8^+^ T-cells and downregulated in highly potent *Transcription Factor 7* (*TCF7*; encoding T cell factor 1, TCF1)-expressing CD8^+^ T-cells, may potentiate CAR T-cell stemness and antitumor function. This hypothesis is further supported by the results of a very recently published study showing that genetic knockout of *PRDM1* in T-cells enhances persistence and therapeutic responses in cancer models of adoptive immunotherapy (23). Accordingly, *PRDM1* single knockout (KO) CAR T-cells exhibited enrichment of the *TCF7*-expressing CD8^+^ phenotype in association with improved CAR T-cell persistence and expansion. However, genetic deletion of *PRDM1* alone resulted in only marginal enhancement of CAR T-cell efficacy against B-cell leukemia and completely failed to rescue CAR^+^ tumor-infiltrating lymphocyte (TIL) function in animal models of high tumor burden. These results are consistent to some degree with previous reports of antigen-specific *PRDM1* KO T-cells exhibiting comparable or even inferior control of viruses and cancer through mechanisms that remain largely elusive (24–27). We found that during prolonged tumor antigen exposure, several exhaustion-related transcription factors, including Nuclear Receptor Subfamily 4 Group A Member 3 (NR4A3), were reciprocally upregulated in *PRDM1*-deficient CAR T-cells and drove epigenetically programmed T-cell dysfunction in a Nuclear factor of activated T-cells (NFAT)-dependent manner. *PRDM1* and *NR4A3* dual-ablation counteracted the development of T-cell exhaustion, which was not observed with single KO of either gene. This strategy also led to a marked increase in the survival and expansion of TILs; increased the capacity of the CAR TILs to produce effector cytokines after stimulation; and reduced their expression of inhibitory factors. In immunodeficient mice bearing soft tissue or bone engrafted prostate cancer cells, pancreatic tumors or aggressive B-cell leukemia, a single intravenous infusion of *PRDM1*/*NR4A3* dual KO CAR T-cells resulted in higher antitumor efficacy, T-cell response durability upon rechallenge and overall prolongation of survival compared to conventional CAR T-cell treatment. There is considerable interest in modulating CAR T-cells to control tumors more successfully. Our identification of the reciprocal nature of BLIMP1 and NR4A3 activity as a distinct mechanism regulating the balance of T-cell differentiation and function represents an actionable and therapeutically effective strategy for achieving this desired outcome.

## Results

### *TCF7*- and *HAVCR2*-expressing CD8^+^ populations within infusion products are associated with favorable and poor CAR T-cell-mediated therapeutic potency, respectively.

**Figure 1.**
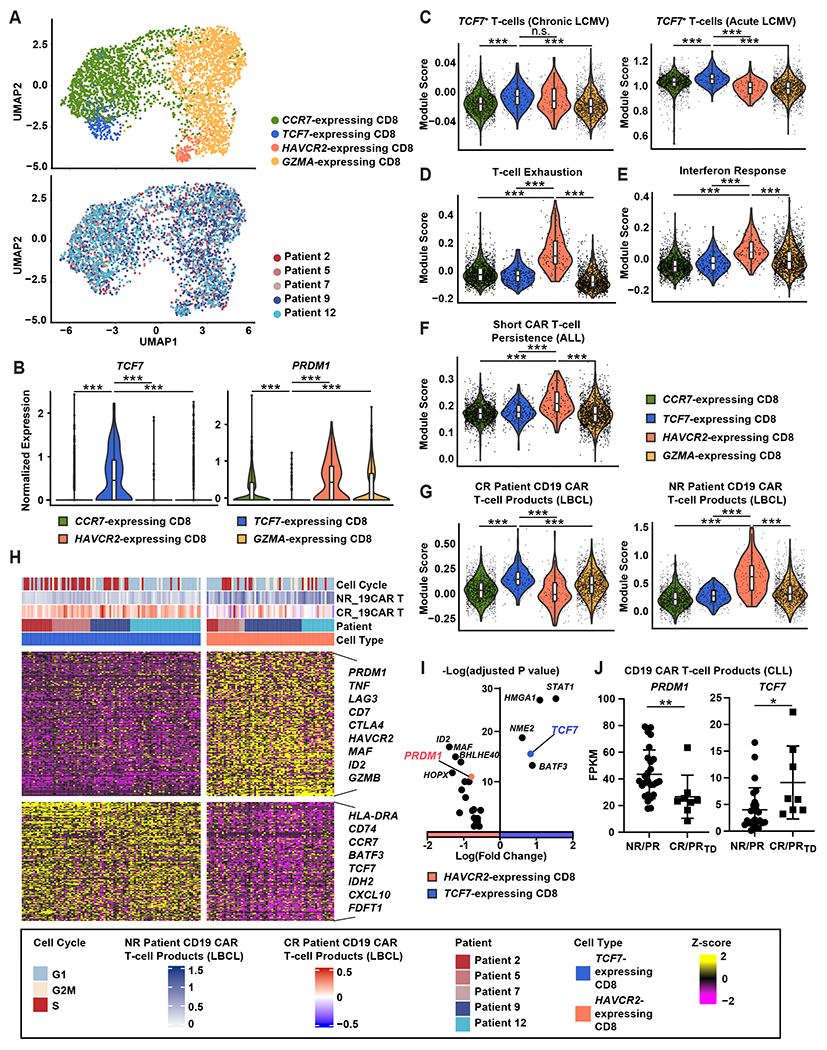
*TCF7*- and *HAVCR2*-expressing CD8^+^ populations within infusion products are associated with favorable and poor CAR T-cell therapeutic potency, respectively. (**A**) Uniform manifold approximation and projection (UMAP) plots showing sub-clustering of CD8^**+**^ T-cells from CAR T-cell infusion products for patients with mCRPC. Cells are labeled according to marker gene expression and patient origin. (**B**) Expression of *PRDM1* and *TCF7* is shown for CD8^+^ T-cell subclusters. (**C to E**) Scores of gene signatures enriched in (**C**) *TCF7*-expressing T-cells in LCMV clone 13 (GSE83978; left) and LCMV Armstrong infection (GSE83978; right), (**D**) exhausted T-cells (GSE136796) and (**E**) the IFN response (dbGaP phs002323.v1.p1) are shown. (**F and G**) Shown are CD8^+^ T-cell subcluster-specific gene signature scores enriched in (**F**) pre-manufactured T-cells from patients with ALL with poor CD19 CAR T-cell persistence (dbGaP phs002323.v1.p1) and (**G**) anti-CD19 CAR T-cell infusion products of complete responder patients (CR) and non-responder (NR) patients with LBCL (GSE151511). (**H**) Differentially expressed genes were compared between *TCF7*- and *HAVCR2*-expressing CD8^+^ clusters. Top bars indicate cell clusters, patient origin, CD19 CAR T-cell response score (GSE151511), and cell cycle. (**I**) Differential expression of transcription factors between *TCF7*- and *HAVCR2*-expressing CD8^+^ clusters is shown. (**J**) *PRDM1* and *TCF7* expression was evaluated in CD19 CAR-T infusion products from patients with chronic lymphocytic leukemia (CLL; CR: complete response; PR_TD_: very good partial response; PR: partial response; NR: no response); FPKM: Fragments per kilo base of transcript per million mapped fragments. *P < 0.05, **P < 0.01, ***P < 0.001, n.s., not significant as measured by Kruskal-Wallis test with a post-hoc Dunn’s multiple comparison test. Violin plots in (**B**) to (**G**) indicate the distribution of data with rectangles in the middle of the density curves showing the ends of the first and third quartiles and central horizontal line depicting the median. Horizontal bars in (**J**) show the mean.

We retrospectively performed scRNA-seq analysis on five autologous PSMA CAR T-cell infusion products administered to patients with mCRPC (fig. S1A). These products were selected based on sample availability and to represent a range of outcomes including *in vivo* CAR T-cell expansion and prostate-specific antigen (PSA) response (22). 20,702 cells were obtained after eliminating low-quality cells, consisting mostly of T-cells and rare B-cells (fig. S1B and C). To identify transcriptional features associated with CAR T-cell therapeutic potency, we focused on CD8^+^ T-cells. Clustering all CD8^+^ T-cells revealed four major cell states: early memory-like *TCF7*- and *C-C Motif Chemokine Receptor 7* (*CCR7*)-expressing CD8^+^ clusters; effector-like *Granzyme A* (*GZMA*)- and *HAVCR2*-expressing CD8^+^ sub-populations (Fig. 1A, fig. S1D).

A *TCF7*-expressing CD8^+^ subset was the only cell population that expressed a high abundance of *TCF7* (Fig. 1B, fig. S1D). In accordance with this *TCF7* expression profile, these clusters were enriched in *TCF7* stem cell-like T-cell signatures that are associated with robust antiviral responses in mouse models of acute and chronic lymphocytic choriomeningitis virus (LCMV) infection (Fig. 1C) (28–30). In contrast, a *HAVCR2*-expressing CD8^+^ cluster was markedly enriched in T-cell exhaustion signatures, whereas *CCR7*- and *TCF7*-expressing CD8^+^ populations possessed low exhaustion scores (Fig. 1D). Consistent with this analysis, the *HAVCR2*-expressing CD8^+^ T-cell population exhibited upregulation of multiple inhibitory molecule transcripts compared to *TCF7*-expressing CD8^+^ cells (fig. S1E).

A recent study that characterized leukapheresed T-cells of pediatric patients with acute lymphocytic leukemia (ALL) demonstrated that enrichment of an interferon (IFN) response signature is associated with poor CAR T-cell persistence (31). We thus queried the CD8^+^ T-cell clusters identified in our analysis for this battery of genes and found that *HAVCR2*-expressing CD8^+^ T-cells in mCRPC patient infusion products had significantly (*P* < 0.001) higher IFN response signature scores compared to the other populations (Fig. 1E). Accordingly, the *HAVCR2*-expressing CD8^+^ state is associated with short CD19 CAR T-cell persistence in ALL, which often prognosticates poor outcome (Fig. 1F).

To determine the generalizable nature of our initial findings, we next sought to investigate CD8^+^ clusters associated with response to CAR T-cell therapy in LBCL (21). Intriguingly, the *TCF7*-expressing CD8^+^ population highly expressed a gene set enriched in anti-CD19 CAR T-cell infusion products derived from complete responders (CR), whereas the *HAVCR2*-expressing CD8^+^ T-cell population exhibited the highest scores for a gene set that characterizes non-responder (NR) anti-CD19 CAR T-cells (Fig. 1G). These results were concordant with exhaustion signatures in other independent data sets (fig. S1F). We then scored PSMA CAR T-cell infusion products for the *TCF7* and *HAVCR2* profiles and analyzed how these signature scores correlate with PSA decline in patients with mCRPC following CAR T-cell transfer (fig. S1G and H). In accordance with our recently reported clinical findings (22), there is a clear dose- and lymphodepletion-dependent relationship with peripheral blood CAR T-cell expansion and early antitumor effects, as determined by serum PSA decrease (fig. S1H). Patients 2 and 5 were treated in the cohort that received the lowest dose of CAR T-cells in this trial, without lymphodepleting preconditioning prior to cell infusion. It is intriguing that, independently of conditioning and cell dose, Patient 2 pre-infusion CAR T-cells with a highly enriched *TCF7* signature and a low *HAVCR2* score exhibited superior in vivo expansion and PSA response induction, compared to the infusion product of Patient 5 with lower *TCF7* and elevated *HAVCR2* population scores (fig. S1G and H). These results suggest that increasing *TCF7*-expressing CD8^+^ populations and depleting *HAVCR2*-expressing populations in CAR T-cell infusion products may enhance CAR T-cell efficacy.

Among the transcription factors upregulated in the *HAVCR2*-expressing CD8^+^ population, we focused on BLIMP1, which is encoded by *PRDM1* (Fig. 1H and I). *PRDM1* is known to play a central role in driving T-cell exhaustion and terminal differentiation (27, 32, 33). In addition, high expression of *PRDM1* is associated with loss of T-cell stemness and self-renewal capacity through repression of *TCF7* (34). Consistent with these results, *HAVCR2*-expressing CD8^+^ cells displayed the highest expression of *PRDM1*, whereas *TCF7*-expressing CD8^+^ T-cells exhibited low expression of *PRDM1* among the CD8^+^ clusters (Fig. 1B). To further validate these findings, we compared *PRDM1* and *TCF7* expression in cohorts of patients with chronic lymphocytic leukemia (CLL) treated with autologous CD19 CAR T-cells. As expected, pre-infusion CAR T-cells from CR and very good partially responding (PR_TD_) individuals exhibited significantly lower expression of *PRDM1* (*P* < 0.01) and elevated *TCF7* (*P* < 0.01), compared to poorly functional products from non-responders (NR) or conventional partial responders (PR) (Fig. 1J).

The frequencies of CD4^+^ and CD8^+^ T-cells within the clinical CAR infusion products profiled in this study are presented in fig. S2A. scRNA-seq analysis of the CD4^+^ compartment was also performed (fig. S2B to D). Clustering all CD4^+^ T-cells revealed four major cell states: *TCF7*-, *CCR7*-, *Marker of Proliferation Ki-67* (*MKI67*) and *Cytotoxic T-Lymphocyte Associated Protein* 4 (*CTLA4*)-expressing CD4^+^ clusters. *CTLA4*-expressing CD4^+^ cells displayed the highest expression of *PRDM1* whereas *TCF7*-expressing CD4^+^ T-cells exhibited low *PRDM1* among the CD4^+^ clusters (fig. S2D). We therefore originally hypothesized that *PRDM1* mediates CAR T-cell exhaustion and attrition of stemness. Thus, *PRDM1* KO may mitigate T-cell exhaustion and improve CAR T-cell expansion, persistence and antitumor efficacy.

### CRISPR/Cas9-mediated *PRDM1* KO potentiates early memory differentiation of PSMA CAR T-cells.

**Figure 2.**
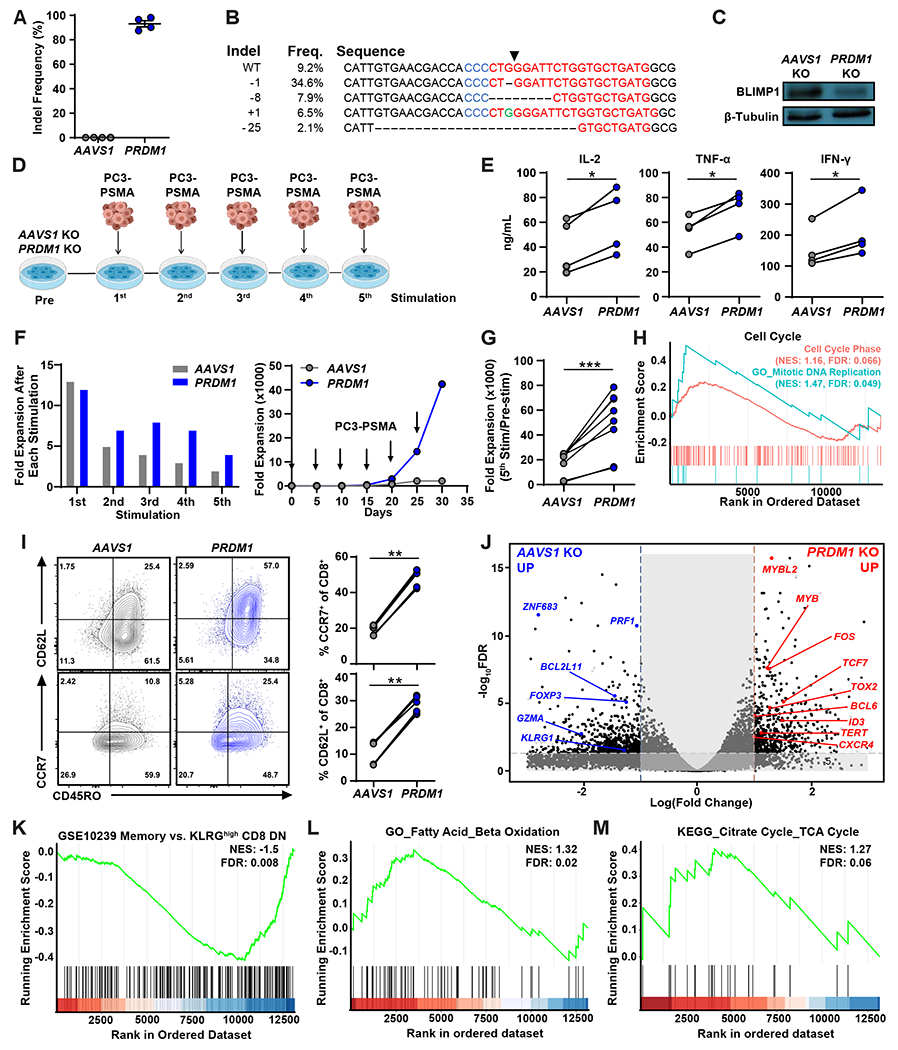
CRISPR/Cas9-mediated PRDM1 KO potentiates early memory PSMA CAR T-cell differentiation. (**A**) *PRDM1* editing efficiency was measured by Sanger sequencing and subsequent TIDE (Tracking of Indels by Decomposition) analysis. Data are presented as scatter points, where the mean and S.E.M. bars are included. (**B**) Amplicon sequencing of *PRDM1* indel variants generated by CRISPR/Cas9-mediated gene editing is shown. Arrow indicates cleavage site. (**C**) Representative Western blot analysis for BLIMP1 expression is shown. (**D**) Schematic of the restimulation assay used to “stress test” *PRDM1* KO PSMA CAR T-cells. CAR T-cells were challenged with PSMA-expressing PC3 prostate tumor targets every 4 to 5 days at an effector to target (E:T) ratio of 3:1. (**E**) Effector cytokines produced by CAR T-cells after the initial tumor cell challenge. (**F**) Representative CAR T-cell expansion kinetics during the restimulation assay for one donor are shown. Left: CAR T-cell expansion after each stimulation, Right: Cumulative CAR T-cell expansion. Arrows indicate the timing of PC3-PSMA tumor cell challenge. (**G**) Summary of the expansion capacity of *AAVS1* and *PRDM1* KO CAR T-cells during the restimulation assay with four different donors. (**H**) Gene set enrichment analysis (GSEA) of *PRDM1* KO versus *AAVS1* KO CAR T-cells comparing gene signatures related to the cell cycle and mitotic DNA replication. CAR-T samples were harvested on day 5 following the first tumor challenge. NES: normalized enrichment score, FDR: false discovery rate. (**I**) Early memory marker expression was measured by flow cytometry after two consecutive tumor cell stimulations. (**J**) Volcano plot displaying the results of differential gene expression analysis when comparing *PRDM1* KO to control *AAVS1* KO CAR T-cells. (**K to M**) GSEA of *PRDM1* KO versus *AAVS1* KO CAR T-cells comparing gene sets associated with (**K**) memory T-cells (GSE10239), (**L**) GO_Fatty acid_Beta oxidation, and (**M**) the KEGG_TCA cycle. All knockout and restimulation experiments were conducted with CAR T-cells manufactured from 4 different healthy donors. RNA-seq experiments were conducted with CAR T-cells manufactured from 2 different healthy individuals, each with replicates generated from two independent experiments. *P < 0.05, **P < 0.01, ***P < 0.001, as measured by paired *t*-tests.

To examine how BLIMP1 deficiency affects CAR T-cell fate, we knocked out *PRDM1* in PSMA CAR T-cells using Clustered Regularly Interspaced Short Palindromic Repeats (CRISPR)/CRISPR-associated protein 9 (Cas9) technology (Fig. 2A to C). Because the dominant negative transforming growth factor (TGF)-β receptor component used in our recently published clinical study (22) can potentially confound findings, this was not included in the tested constructs. CAR T-cells with KO at the *Adeno-associated virus integration site 1* (*AAVS1*) safe harbor locus were used as a negative control for gene editing in subsequent in vitro and in vivo functional studies (35). PSMA CAR expression was comparable between control *AAVS1* KO and *PRDM1* KO CAR T-cells (fig. S3). Next, we characterized the phenotype and function of *PRDM1* KO CAR T-cells during an in vitro “stress test” involving repetitive PSMA stimulation through the CAR (Fig. 2D). *PRDM1* KO CAR T-cells initially showed increased effector cytokine production compared to control CAR T-cells at 24 hours after the first restimulation (Fig. 2E). In addition, we found that, whereas the expansion capacity of *AAVS1* KO CAR T-cells gradually declined following repetitive stimulation, *PRDM1* KO CAR T-cells sustained high proliferative capacity even after multiple rounds of stimulation and concomitantly upregulated cell cycle-related gene signatures (Fig. 2F to H). This enhanced expansion capacity of *PRDM1* KO CAR T-cells is likely attributed to increased memory formation. Accordingly, *PRDM1* KO CAR T-cells displayed increased expression of *CD62L*, *CCR7*, *MYB*, *ID3*, and *TCF7* as well as enriched transcriptomic signatures of memory precursor effector cells, fatty acid oxidation, and the tricarboxylic acid cycle, indicating that *PRDM1* KO CAR T-cells are skewed toward an early memory fate (Fig. 2I to M).

### *PRDM1* KO increases *TCF7* expression and enhances early memory CAR T-cell differentiation in a *TCF7*-dependent manner.

**Figure 3.**
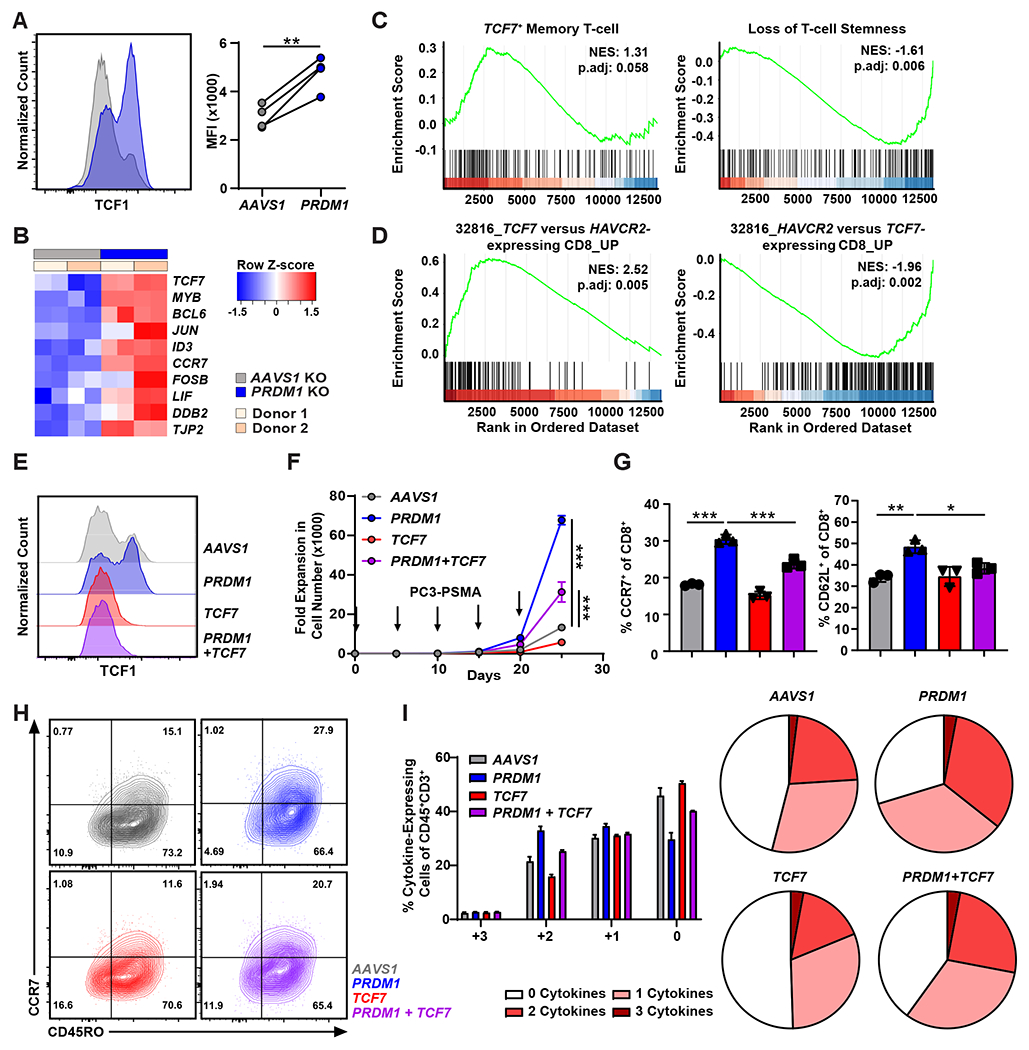
*PRDM1* KO increases *TCF7* expression and enhances early memory CAR T-cell differentiation in a *TCF7*-dependent manner. (**A**) TCF1 expression was measured by flow cytometric analysis of *PRDM1* KO versus *AAVS1* KO PSMA CAR T-cells. CAR T-cells derived from *n* = 4 different healthy individuals. MFI: mean fluorescence intensity. (**B**) Analysis of CAR T-cells for transcripts enriched in *TCF7*-expressing stem cell-like T-cells previously observed in LCMV mouse models (GSE83978). (**C**) GSEA of *PRDM1* KO relative to *AAVS1* KO CAR T-cells comparing gene sets associated with a *TCF7*^+^ T-cell memory state (GSE83978) and loss of stemness (GSE84105). p.adj, adjusted *P* value. (**D**) GSEA of *PRDM1* KO versus *AAVS1* KO CAR T-cells evaluating gene sets enriched in *TCF7*^+^ (left) and *HAVCR2*^+^ CD8^+^ clusters (right). Gene signatures were derived from CAR T-cell infusion products of patients with mCRPC shown in Figure 1. (**E**) Representative histogram shows TCF1 expression by flow cytometry in *PRDM1* and *TCF7* KO CAR T-cell variants. (**F**) Expansion kinetics are shown during a restimulation assay of gene-edited CAR T-cells. CAR T-cells were challenged with PC3-PSMA target cells every 5 days at an E:T of 3:1. Arrows indicate the timing of PC3-PSMA cell restimulation. Data represent the mean ± S.D. (*n* = 3 independent experiments). (**G**) Frequencies of CAR T-cell variants expressing CCR7 and CD62L and (**H**) representative flow cytometry plots showing CCR7 and CD45RO expression following the first in vitro tumor cell challenge. Data indicate mean ± S.D. from *n* = 3 independent experiments. (**I**) CAR T-cell polyfunctionality (IL-2, IFN-γ and TNF-α expression) was evaluated after a 15-hour co-culture with PC3-PSMA cells. Data show mean ± S.D. from *n* = 3 independent experiments. *P < 0.05, **P < 0.01, ***P < 0.001. Data in panel (**A**) and panels (**F** and **G**) were analyzed using a paired *t*-test and one-way ANOVA test with a post-hoc Tukey’s multiple comparison test, respectively.

We next sought to investigate whether *PRDM1* KO derepresses *TCF7* expression. *PRDM1* KO increased expression of *TCF7* and genes encoding other transcription factors that are crucial for maintaining T-cell stemness, such as Myeloblastosis (MYB), B-cell lymphoma 6 (BCL6) and Inhibitor of DNA-binding 3 (ID3) (Fig. 3A and B) (36–38). Moreover, *PRDM1* KO CAR T-cells were enriched in *TCF7*-expressing stem cell-like T-cell signatures, suggesting that BLIMP1 inhibits *TCF7*-mediated stemness (Fig. 3C). Consistent with our hypothesis, *PRDM1* KO CAR T-cells exhibited depletion of *HAVCR2*-expressing CD8^+^ cell population gene signatures and transcriptionally resembled *TCF7*-expressing CD8^+^ T-cells observed in PSMA CAR T-cell infusion products (Fig. 3D). To examine whether *TCF7* upregulation is required for the maintenance of early memory differentiation and robust CAR T-cell proliferative potential as observed in Fig. 2, we knocked out *TCF7* (Fig. 3E) and subsequently assessed CAR T-cell expansion in a restimulation assay. We found that *TCF7* depletion counteracted the effect of *PRDM1* KO by reducing proliferative capacity, which was accompanied by decreased frequencies of CCR7 and CD62L expressing memory CAR T-cells (Fig. 3F to H). Because lack of polyfunctionality is a hallmark of terminal differentiation and T-cell dysfunction (38), we next stimulated *PRDM1* and *TCF7* single or double KO CAR T-cells and measured expression of interleukin (IL)-2, interferon (IFN)-γ and tumor necrosis factor (TNF)-α. *PRDM1* ablation increased frequencies of polyfunctional CAR T-cells compared to *AAVS1* KO control CAR T-cells (Fig. 3I), which coincides with the observed increased early memory differentiation of *PRDM1* KO CAR T-cells. Conversely, *TCF7* depletion decreased the frequency of polyfunctional *PRDM1* KO CAR T-cell populations (Fig. 3I), suggesting that *PRDM1* deficiency enhances CAR T-cell polyfunctionality, at least in part, by *TCF7* upregulation. Taken together, these data imply that *PRDM1* knockout enhances CAR T-cell early memory differentiation in a *TCF7*-dependent manner.

### *PRDM1* KO hampers T-cell effector function and tumor control during chronic CAR stimulation despite increases in CAR T-cell proliferative capacity.

**Figure 4.**
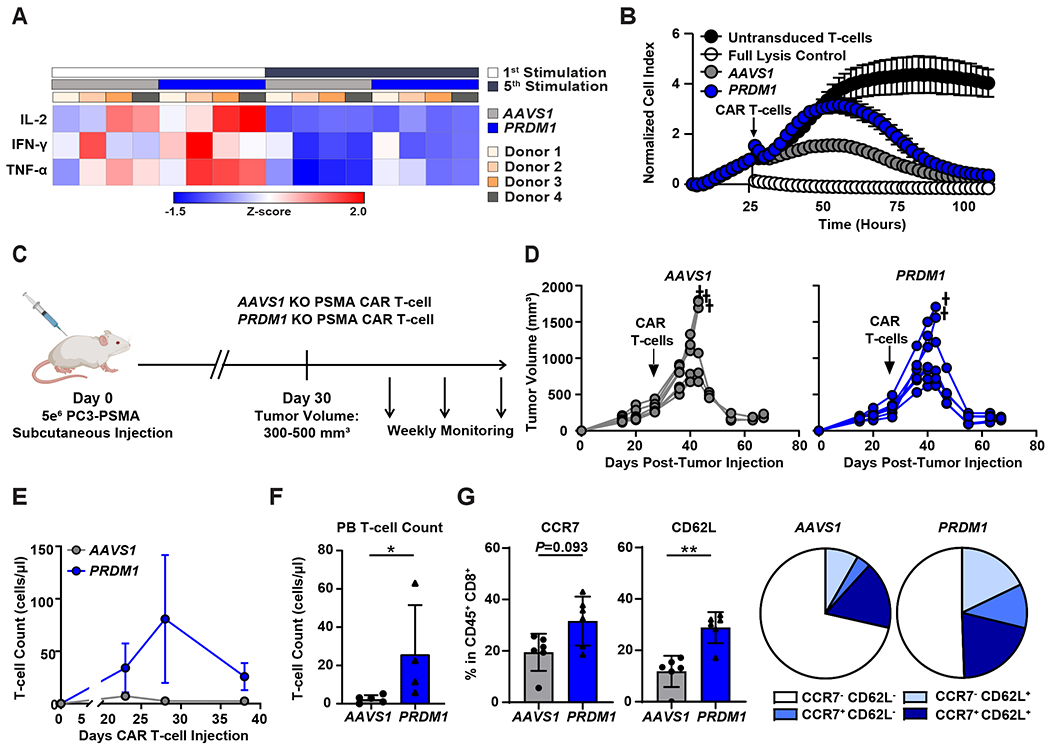
*PRDM1* KO marginally enhances solid tumor control, despite increases in CAR T-cell early memory phenotype and proliferative capacity. **(A and B)** CAR T-cells were restimulated five times with PC3-PSMA target cells every 4 to 5 days at an E:T ratio of 3:1. (**A**) The heat map shows the relative effector cytokine secretion by *AAVS1* KO and *PRDM1* KO CAR T-cells after first and fifth tumor cell restimulations. (**B**) Killing kinetics of *AAVS1* KO and *PRDM1* KO CAR T-cells are shown. CAR T-cells were isolated after the fifth restimulation timepoint and co-cultured with PC3-PSMA cells at a ratio of E:T = 3:1. Cytotoxicity was monitored by real-time cellular impedance monitoring technology (xCELLigence). T-cells without CAR transduction were used as a negative control and 20% Tween-20 treatment served as a full lysis control. Data are expressed as mean ± S.D. from *n* = 6 individual donors. (**C**) A schematic of the high tumor burden PC3-PSMA xenograft mouse model. Male NSG mice were subcutaneously transplanted with 5 × 10^6^ PC3-PSMA cells, and 3.5 × 10^5^ PSMA CAR T-cells were given intravenously when tumor volume reached about 500 mm^3^. (**D**) Tumor growth was monitored by caliper measurements. † = death. (**E**) CAR T-cell expansion kinetics in the peripheral blood are shown. (**F**) Absolute numbers of human T-cells in the peripheral blood on day 38 post-CAR T-cell injection are shown. (**G**) Frequencies of peripheral blood CAR T-cells expressing CCR7 and CD62L were measured by flow cytometry. Data are shown as mean ± S.D.; *n* = 6 for (**D**) and (**G**) and *n* = 4 to 5 for (**E**) and (**F**). *P < 0.05, **P < 0.01, as determined by Mann Whitney U tests.

To evaluate the effector functions of *PRDM1* KO CAR T-cells in the setting of repetitive antigen exposure, we first assessed cytokine concentrations 24 hours after the first and fifth rounds of in vitro PC3-PSMA tumor cell stimulation. *PRDM1* KO initially increased effector cytokine production as shown in Fig. 3I and Fig. 4A. However, after multiple tumor cell stimulations, *PRDM1* KO CAR T-cells exhibited dramatically reduced effector cytokine secretion (Fig. 4A). Further, after five consecutive tumor challenges, the cytolytic activity of PRDM1 KO CAR T-cells was impaired compared to that of control CAR T-cells (Fig. 4B). This result is consistent with a previous study in which *PRDM1* deficiency profoundly compromised the cytotoxic activity of antigen-specific CD8^+^ T-cells during chronic viral infection (32).

Based on the above results, we next assessed the in vivo antitumor activity of *PRDM1* KO CAR T-cells in xenogeneic mouse models. When tested against a relatively low burden of flank-engrafted PC3-PSMA prostate tumor cells, *PRDM1* KO PSMA CAR T-cells exhibited a modest enhancement of tumor control compared to control CAR T-cells (fig. S4A and B). Similarly, and consistent with a recent report (23), *PRDM1* KO anti-CD19 CAR T-cells better suppressed cancer growth compared to control CAR T-cells in a B-cell acute lymphoblastic leukemia (ALL) model (NALM-6), although these CAR T-cells eventually failed to eradicate tumors (fig. S4C to E). In an in vivo “stress test” in which tumor burden is escalated to reveal CAR T-cell functional limits, *PRDM1* KO CAR T-cells showed comparable antitumor activity to *AAVS1* KO CAR T-cells (Fig. 4C and D). Despite lack of improved tumor control over *AAVS1* KO CAR T-cells, *PRDM1* KO CAR T-cells exhibited enhanced in vivo expansion and persistence (Fig. 4E and F). Additionally, consistent with our in vitro studies, *PRDM1* KO CAR T-cells maintained a higher fraction of central memory T-cells (Fig. 4G, fig. S4F), indicating that *PRDM1* deficiency improves CAR T-cell expansion and persistence by preserving early memory pools. Together, these results suggest that, despite improvements in expansion and persistence, *PRDM1* KO alone is not sufficient to potentiate robust and sustained CAR T-cell antitumor efficacy in aggressive tumor models.

### *PRDM1* KO CAR T-cells fail to maintain high effector function due to upregulation of exhaustion-related transcription factors.

**Figure 5.**
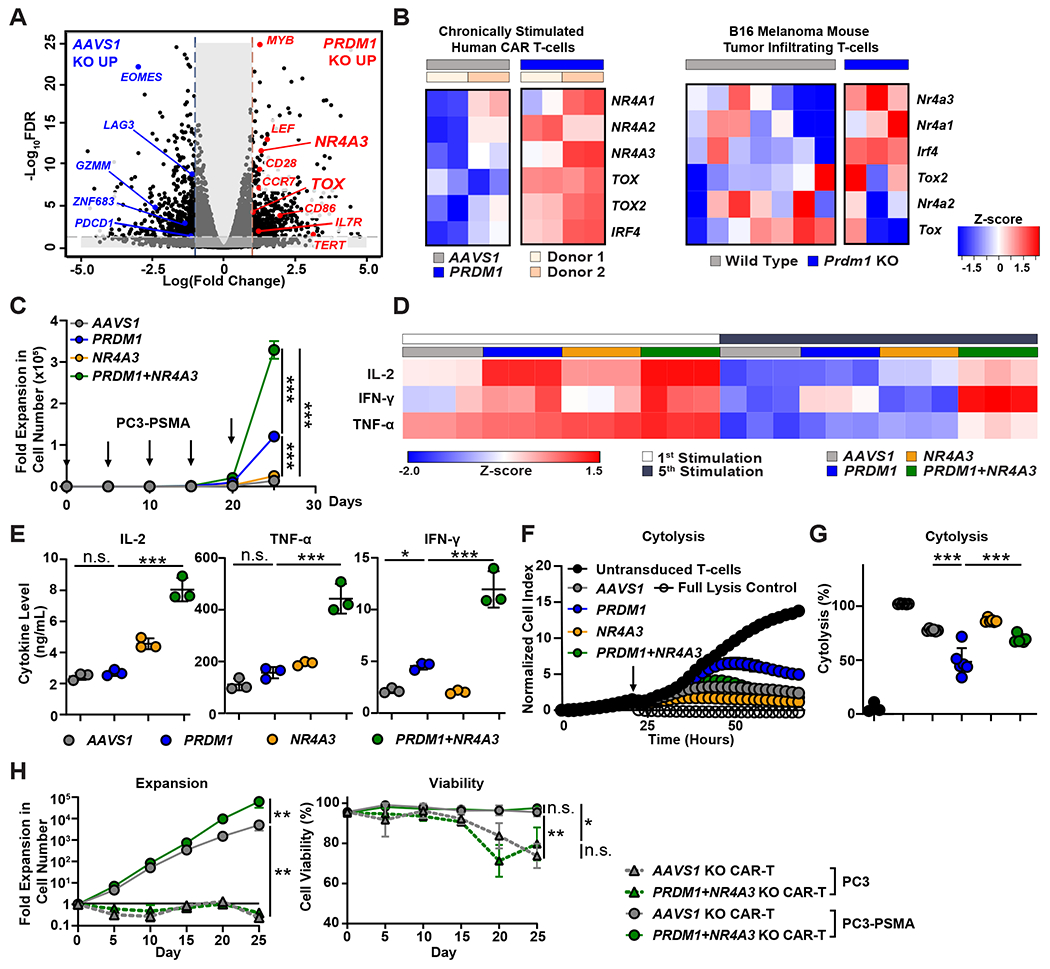
*PRDM1* KO CAR T-cells fail to sustain antitumor effector function due to upregulation of exhaustion-related transcription factors. (**A**) CD8^+^ CAR T-cells were isolated after the fourth tumor restimulation for bulk RNA-seq. The volcano plot illustrates differential gene expression analysis in *PRDM1* KO compared to control *AAVS1* KO CAR T-cells after the fourth consecutive tumor cell challenge. (**B**) The heat map shows expression of transcription factor-encoding genes associated with T-cell exhaustion. RNA-seq experiments were conducted with CAR T-cells manufactured from 2 different individuals, each with replicates generated from two independent experiments (left). A similar profile of exhausted murine TILs (GSE113221) is shown in the right panel. (**C**) Expansion kinetics of *PRDM1* and *NR4A3* KO CAR T-cells during a restimulation assay. PSMA CAR T-cells were challenged with PC3-PSMA tumor cells every 5 days at an E:T ratio of 3:1. Arrows indicate the timing of PC3-PSMA challenge. Data are mean ± S.D. from *n* = 3 different donors. (**D** and **E**) Concentrations of effector cytokines produced by *AAVS1* KO, *PRDM1* KO, *NR4A3* KO and *PRDM1*/*NR4A3* dual KO CAR T-cells are shown. (**D**) The heat map shows effector cytokine secretion 24 hours following the first and fifth tumor cell challenges. (**E**) Graphical summaries are shown for effector cytokine production after the fifth CAR T-cell stimulation with tumor targets. Data are mean ± S.D. (*n* = 3). (**F**) Killing kinetics are shown for the indicated CAR T-cell/tumor cell co-cultures. (**G**) CAR T-cells were isolated after the fifth round of antigen stimulation and co-cultured with PC3-PSMA tumor cells at an E:T ratio of 3:1 for a “stressed” cytotoxicity assay. Data in (**F and G**) indicate mean ± S.D. (*n* = 6). For (**C to G**), data are representative of 3 independent experiments performed with engineered CAR T-cells manufactured from 3 different healthy individuals. (**H**) CAR T-cells were repetitively challenged with PC3 or PC3-PSMA tumor targets every 5 days at an E:T ratio of 3:1. The expansion capacity (left) and viability (right) of CAR T-cells were assessed over time. Data are depicted as the mean ± S.D. from *n* = 3 independent donors assayed in 2 independent experiments. For all panels, *P < 0.05, **P < 0.01, ***P < 0.001, n.s., not significant, as determined by a one-way ANOVA test with a post-hoc Tukey’s multiple comparison test.

To elucidate the mechanism by which *PRDM1* KO CAR T-cells fail to maintain effector function following chronic antigen stimulation, we performed bulk RNA-seq on CAR T-cells harvested after several rounds of tumor challenge. We observed that *PRDM1* KO increased the expression of early memory-related genes, including *MYB*, *LEF*, *CCR7*, *IL7R*, and *CD28*, even after multiple stimulations (Fig. 5A). Intriguingly, together with these early memory-related genes, *PRDM1* KO resulted in upregulation of genes encoding multiple exhaustion-related transcription factors such as the Nuclear Receptor Subfamily 4A (NR4A) transcription factors as well as Thymocyte Selection-associated HMG BOX (TOX), TOX2, and Interferon Regulatory Factor 4 (IRF4; Fig. 5B, **left**). To rule out potential model-dependent effects, we took advantage of a publicly available RNA sequencing expression dataset of CD8^+^ TILs from B16F10 (melanoma) tumor-bearing *PRDM1* conditional knockout (cKO) syngeneic mice. Concordant with our findings in CAR T-cells, *PRDM1* cKO CD8^+^ TILs exhibited upregulated expression of *NR4A3*, *NR4A1*, and *IRF4* compared to wild type counterparts (Fig. 5B, **right**).

### Combinatorial *PRDM1* and *NR4A3* KO sustains the effector function of chronically-stimulated CAR T-cells.

We hypothesized that the observed compensatory upregulation of exhaustion-associated transcription factor genes can limit the effector function of *PRDM1* KO CAR T-cells during chronic stimulation and that deletion of these exhaustion factors will render *PRDM1* KO CAR T-cells capable of maintaining antitumor effector function. *PRDM1* KO CAR T-cells exhibited reduced effector function, despite inhibitory receptor downregulation (fig. S5A). Because *NR4A3* was the most significantly upregulated exhaustion-related transcription factor gene examined in *PRDM1* KO CAR T-cells after multiple episodes of antigen stimulation (*P* < 0.001, Fig. 5B) and *NR4A3* is significantly elevated in hypofunctional NR/PR CLL patient CD19 CAR T-cells (*P* < 0.01, fig. S5B), we next knocked out both *NR4A3* and *PRDM1* (fig. S5C) and functionally characterized the gene-edited CAR T-cells. We observed that, whereas *NR4A3* single KO CAR T-cells exhibited a similar degree of expansion as control CAR T-cells, *PRDM1*/*NR4A3* dual KO CAR T-cells exhibited the highest degree of antigen-induced proliferative capacity (Fig. 5C). We also measured the frequency of CAR T-cells expressing cytotoxic perforin and granzyme molecules. *NR4A3/PRDM1* double KO partially restored cytotoxic function in the setting of perforin and granzyme expression in CD8^+^CAR^+^ T-cells (fig. S5D). *PRDM1*/*NR4A3* KO CAR T-cells also exhibited increased frequencies of CCR7, CD62L, and TCF1 (encoded by *TCF7*) expressing cells compared to *AAVS1* KO CAR T-cells (fig. S5E and F).

Because CD4^+^ CAR T-cells are important to antitumor efficacy (39, 40), we also determined the impact of the various KOs on CD4^+^ CAR T-cells with respect to differentiation phenotypes, as well as cytotoxic molecule expression during the aforementioned in vitro stress tests. Similar to CD8^+^ CAR T-cells, *PRDM1* KO increased frequencies of CD4^+^ CAR T-cells expressing early memory markers such as CCR7 and TCF7 as compared to controls (fig. S5G). Further, combined KO of *PRDM1* and *NR4A3* had a strong effect on rescuing expression of cytotoxic perforin and granzyme molecules in CD4^+^ CAR T-cells (fig. S5H).

Next, we assessed effector functions in the context of *PRDM1*/*NR4A3* KO and found that *PRDM1*/*NR4A3* KO CAR T-cells maintained an elevated abundance of effector cytokine production after multiple tumor challenges, whereas *PRDM1* and *NR4A3* single KO CAR T-cells showed similar IL-2 and TNF-α secretion as control CAR T-cells (Fig. 5D and E). This potency enhancement conferred by *PRDM1* and *NR4A3* dual KO was consistent in the context of both high and low PSMA expression (fig. S5I and J). Furthermore, unlike *PRDM1* single KO CAR T-cells which lost cytotoxic function following serial stimulation, *PRDM1*/*NR4A3* KO CAR T-cells displayed sustained killing activity over time (Fig. 5F and G). To determine whether *PRDM1*/*NR4A3* KO CAR T-cells exhibit aberrant growth patterns potentially indicative of transformation, we tested whether CAR T-cell proliferation is antigen-dependent using irradiated PC3 cells with and without PSMA expression as stimuli. We observed that *PRDM1/NR4A3* KO CAR T-cells fail to expand, accompanied by a reduction in viability when co-cultured with PSMA-negative PC3 cells, suggesting that cell expansion and survival is antigen-dependent (Fig. 5H).

### Upregulation of exhaustion-related transcription factors in *PRDM1* KO CAR T-cells is attributed to increased chromatin accessibility and calcineurin-NFAT signaling.

**Figure 6.**
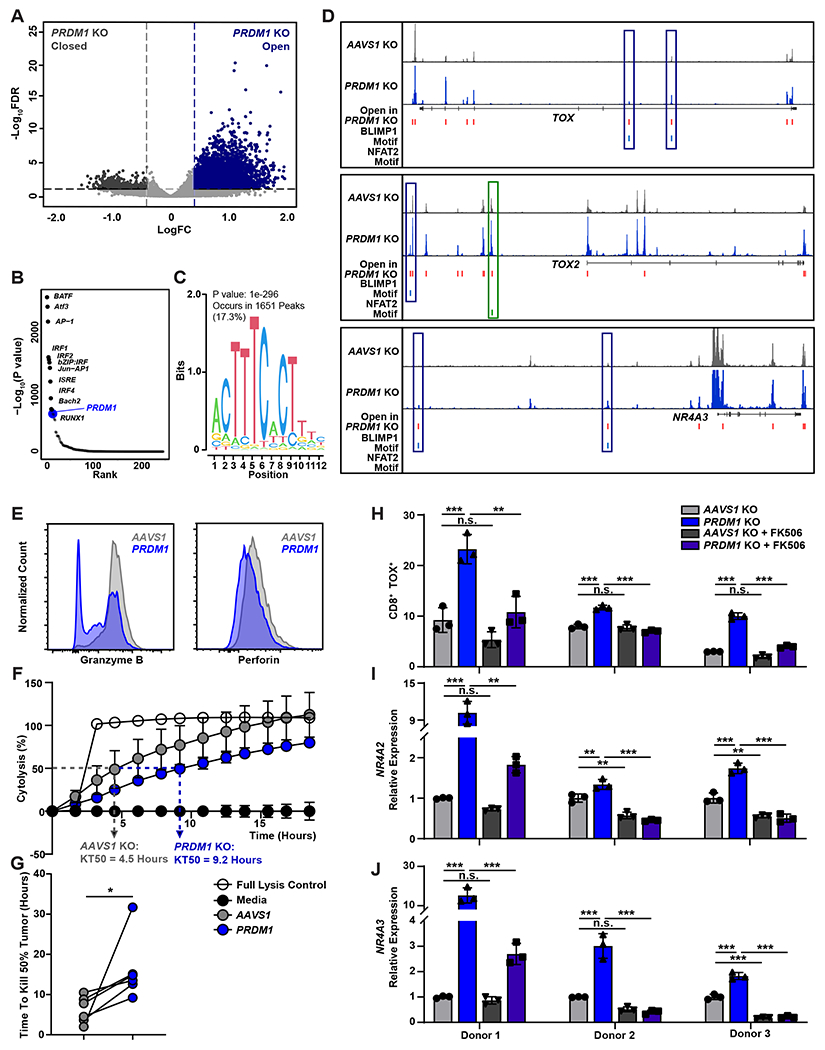
Upregulation of exhaustion-related transcription factors in *PRDM1* KO CAR T-cells is attributed to increased chromatin accessibility and calcineurin-NFAT signaling. (**A** to **D**) ATAC-seq analysis of *AAVS1* KO and *PRDM1* KO CAR T-cells is shown. At the end of manufacturing, CAR^+^ T-cells were enriched and subjected to ATAC-seq analysis. (**A**) The volcano plot shows differentially accessible chromatin regions. (**B**) Top transcription factor motifs enriched in *PRDM1* KO compared to *AAVS1* KO CAR T-cells are shown. (**C**) A BLIMP1 binding motif enriched in open chromatin regions of *PRDM1* KO CAR T-cells is shown. The *P* value was calculated by HOMER motif analysis. (**D**) ATAC–seq tracks of *TOX*, *TOX2*, and *NR4A3* loci are shown. Open chromatin regions in *PRDM1* KO CAR T-cells and the binding motifs of BLIMP1 and NFAT2 are indicated. ATAC-seq experiments were conducted with CAR T-cells manufactured from 2 different donors, each with replicates generated from two independent experiments. (**E**) Expression of granzyme B and perforin were measured by flow cytometry. (**F** and **G**) A cytotoxicity assay was used to determine the time needed to kill 50% of tumor target cells (KT_50_). (**F**) Representative killing kinetics of *AAVS1* and *PRDM1* KO CAR T-cells are shown. Data show mean ± S.D. (*n* = 3). (**G**) KT_50_ values were compared between *AAVS1* and *PRDM1* KO CAR T-cells. Data were generated from 6 independent experiments with CAR T-cells manufactured from 4 different donors. (**H to J**) Expression of exhaustion-related TOX, *NR4A2* and *NR4A3* following repetitive tumor cell challenges is shown. *AAVS1* KO and *PRDM1* KO CAR T-cells were challenged with PC3-PSMA cells every 2 to 4 days at an E:T ratio of 1:1 in the presence or absence of 100 nM FK506. Following two consecutive rounds of stimulation, CAR T-cells were isolated and expression of exhaustion-associated transcription factors or corresponding genes were measured. (**H**) TOX expression was measured by flow cytometry. (**I**) *NR4A2* and (**J**) *NR4A3* expression were measured by qRT-PCR. Data indicate mean ± S.D. (*n* = 3). *P < 0.05, *P < 0.01, ***P < 0.001, n.s., not significant. Data in (**G**) and (**H to J**) were analyzed using a paired *t*-test and one-way ANOVA test with a post-hoc Tukey’s multiple comparison test, respectively.

We next sought to investigate the mechanism of exhaustion-associated transcription factor upregulation in *PRDM1* KO CAR T-cells. Emerging evidence indicates that *PRDM1* epigenetically regulates the transcription of memory-related genes by directly binding to promoter regions and recruiting histone modifiers (41). We therefore investigated whether *PRDM1* KO affects the chromatin accessibility of exhaustion-related transcription factor genes by performing Assay for Transposase-Accessible Chromatin with sequencing (ATAC–seq) on control and *PRDM1* KO CAR T-cells. *PRDM1* KO increased global chromatin accessibility (Fig. 6A). Transcription motif enrichment analysis revealed that a BLIMP1 motif was one of the most enriched transcription factor binding sites in *PRDM1* KO CAR T-cells (Fig. 6B and C), suggesting that BLIMP1 can act as an epigenetic repressor. Consistent with previous chromatin immunoprecipitation sequencing (ChIP-seq) results (41), *PRDM1* KO increased chromatin accessibility at loci corresponding to early memory and stemness genes such as *TCF7*, *CD28*, *CCR7*, *SELL*, and *MYB* (fig. S6A). We found that, even before tumor challenge, *PRDM1* KO increased chromatin accessibility at exhaustion-related transcription factor gene regions, including those of *TOX*, *TOX2*, and *NR4A3*, and that a subset of these open regions colocalized with the above BLIMP1 motif (Fig. 6D). These observations indicate that *PRDM1* contributes to epigenetic repression of a battery of exhaustion-associated transcription factors, and that *PRDM1* KO CAR T-cells are predisposed to upregulate exhaustion-associated transcription factors.

Given that CAR T-cell dysfunction can be induced by prolonged exposure to cancer cells due to low cytotoxic activity (42), we investigated how the impaired cytotoxicity of *PRDM1* KO CAR T-cells affects regulation of exhaustion-associated transcription factors. *PRDM1* KO CAR T-cells downregulated Granzyme B and Perforin expression (Fig. 6E, fig. S5D and H). This decrease in cytotoxic protein expression led to delayed tumor clearance, which caused *PRDM1* KO CAR T-cells to be exposed to target cancer cells for twice as long as control CAR T-cells (Fig. 6F and G). Due to prolonged exposure to cancer cells, *PRDM1* KO increased expression of NFAT2 (fig. S6B). As TOX and NR4A transcription factor families are induced by calcineurin-NFAT signaling, we used the FK506 calcineurin inhibitor to examine the involvement of NFAT in exhaustion-related transcription factor upregulation in *PRDM1* KO CAR T-cells (43, 44). FK506 treatment either completely or partially counteracted *PRDM1* KO-mediated upregulation of TOX protein expression, as well as *NR4A2* and *NR4A3* transcription, during restimulation, suggesting that *PRDM1* KO may induce upregulation of exhaustion-associated transcription factors through increased NFAT signaling (Fig. 6H to J).

### *PRDM1/NR4A3* dual KO enhances in vivo CAR T-cell antitumor activity by preserving TCF1^+^ CD8 T-cells and increasing effector functions.

**Figure 7.**
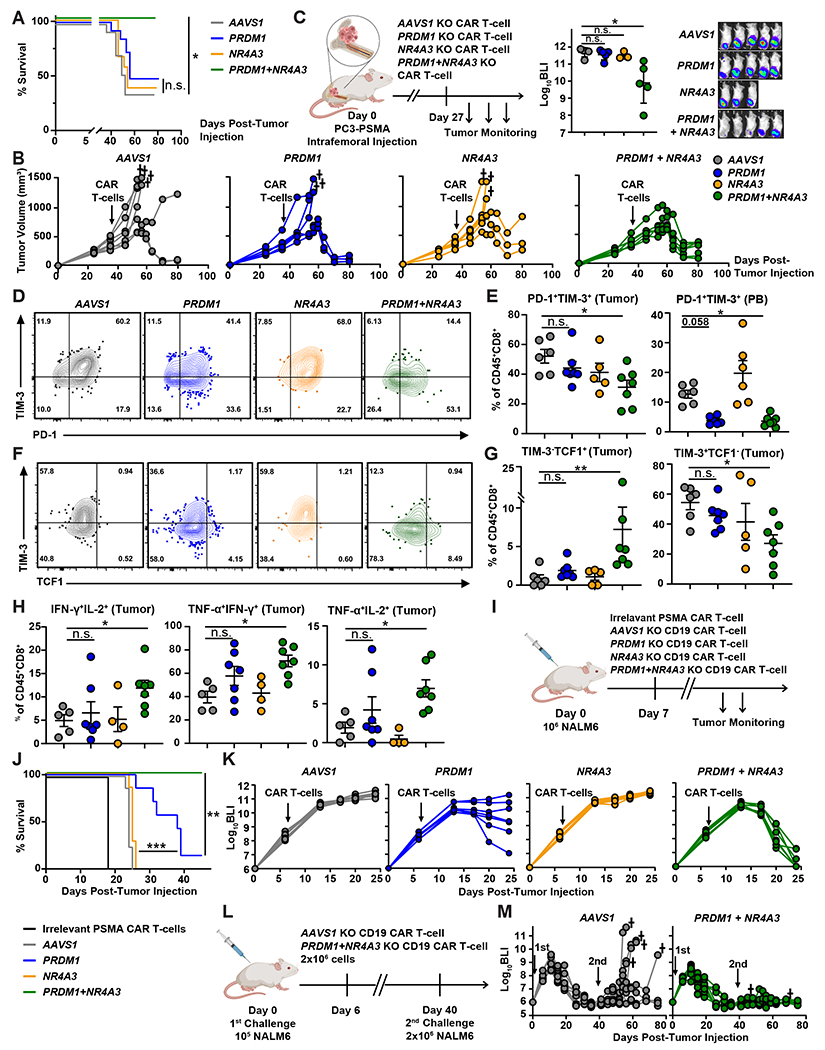
*PRDM1*/*NR4A3* dual KO enhances in vivo CAR T-cell antitumor activity by preserving TCF1^+^CD8^+^ T-cells and increasing effector function. (**A and B**) Male NSG mice were subcutaneously engrafted with 5 × 10^6^ PC3-PSMA tumor cells, and 3.5 × 10^5^ PSMA CAR T-cells were given intravenously when tumor volume reached about 500 mm^3^. (**A**) Kaplan–Meier curves showing overall survival in each group. The Gehan-Breslow-Wilcoxon test was used for statistical analysis (*n* = 12 to 14 mice per group). (**B**) Tumor growth was monitored over time in a representative experiment. (**C**) Male NSG mice were intrafemorally injected with 2 × 10^5^ PC3-PSMA tumor cells. On day 27, 1 to 2 × 10^5^ PSMA CAR T-cells were injected intravenously, and tumor burden was measured by bioluminescent imaging (BLI, (p/sec/cm^2^/sr)). (**D to H**) Tumor and peripheral blood (PB) samples were harvested from mice injected subcutaneously with PC3-PSMA tumor cells on day 45 post-tumor implantation, when the tumor size was comparable between the groups. Samples were stained with hCD45, murine CD45 (mCD45), CD4, and CD8 antibodies and analyzed by flow cytometry. (**D)** Representative flow cytometry plots (**E**) and quantification show frequencies of cells expressing PD-1 and TIM-3 in CD45^+^CD8^+^ T-cells derived from PB and tumors. Representative flow cytometry plots in (**D**) show a tumor sample. (**F**) Representative flow cytometry plots and (**G)** quantification show frequencies of TIM-3- and TCF1-expressing CD45^+^CD8^+^ T-cells derived from tumors. (**H**) Effector cytokine expression was measured by flow cytometry following ex vivo stimulation of CAR TILs. TILs were activated with 50 ng/mL PMA and 1 μg/mL ionomycin in presence of 5 μg/mL Brefeldin A for 6-hours, followed by IFN-γ and TNF-α staining (*n* = 4 to 7). Statistical analysis in (**C, E, G, and H**) was conducted using a Kruskal-Wallis test with a post-hoc Dunn’s multiple comparisons test, *P < 0.05, **P < 0.01, n.s., not significant; mean ± S.E.M. shown. (**I**) A schematic of the NALM-6 xenograft model. Briefly, NSG mice were intravenously injected with 1 × 10^6^ NALM6-CBG cells. On day 7 post tumor injection, 3 × 10^5^ gene-edited anti-CD19 or control CAR T-cells were treated (*n* = 7 to 8 per group). (**J**) survival and (**K**) graphical summaries of longitudinal bioluminescent tumor burden are shown for NSG mice treated, as indicated. Data are representative of two independent experiments. The Gehan-Breslow-Wilcoxon test was used for survival analysis shown in (**J**). (**L**) A schematic of the NALM-6 rechallenge model is shown. Briefly, NSG mice were intravenously injected with 1 × 10^5^ NALM6-CBG cells. On day 6 post-tumor injection, 2 × 10^6^ anti-CD19 CAR T-cells were infused (*n* = 9 to 10 per group). Surviving mice were rechallenged with a second dose of NALM-6 cells on day 40. (**M**) Longitudinal tumor burden is shown.

Based on the observation that *PRDM1/NR4A3* dual KO sustains the proliferative ability, effector functions, and early memory phenotype of CAR T-cells, we next examined whether *PRDM1/NR4A3* KO CAR T-cells would elicit enhanced antitumor activity in vivo. Whereas *AAVS1*, *PRDM1*, and *NR4A3* single KO CAR T-cells controlled tumor growth in about 50% of mice in the high-burden PC3-PSMA cell xenograft model, *PRDM1/NR4A3* double KO CAR T-cells successfully suppressed tumor growth in all treated mice, in association with overall prolongation of survival (Fig. 7A and B). Because advanced prostate cancer commonly progresses within two years following the initiation of androgen-ablative therapy often with osseous in addition to visceral metastases, we also tested our approach in an intraosseous PC3-PSMA prostate tumor model. Bioluminescent tumor burden was also significantly decreased by a single infusion of *PRDM1/NR4A3* KO PSMA CAR T-cells compared to *AAVS1* KO (*P* < 0.05) CAR T-cells in mice bearing intraosseous tumors generated by PC3-PSMA cell engraftment (Fig. 7C). We subsequently collected tumor and blood samples to characterize the immunophenotype of CAR T-cells. We found that *PRDM1/NR4A3* double KO significantly increased the absolute numbers of CAR T-cells in the tumor (*P* < 0.05) and peripheral blood (*P* < 0.001) compared to *AAVS1* KO CAR T-cells fig. S7A). This increased T-cell number may be due in part to the elevated frequencies of early memory T-cells with optimal proliferative potential observed with *PRDM1* KO (fig. S7B), which is consistent with our previous results. To investigate how *PRDM1* and *NR4A3* depletion affect T-cell dysfunction, we first evaluated the frequencies of inhibitory receptor-expressing CAR T-cells. Although *PRDM1* single KO CAR T-cells demonstrated reduced Programmed Death-1 (PD-1) and TIM-3 expression in the peripheral blood, their inhibitory receptor expression was comparable to that of *AAVS1* KO CAR T-cells in the tumor where CAR T-cells receive persistent antigen stimulation (Fig. 7D and E, fig. S7C). In contrast, *PRDM1/NR4A3* KO CAR T-cells demonstrated a significant reduction in the proportion of PD-1^+^TIM-3^+^ CD8 T-cells in both the tumor (*P* < 0.05) and peripheral blood as compared to *AAVS1* KO CAR T-cells (*P* < 0.01, Fig. 7D and E). Also, as observed in vitro, *PRDM1* KO led to a substantial increase in TCF1 (encoded by *TCF7*) expression in CAR T-cells in vivo. Both *PRDM1* single KO and *PRDM1/NR4A3* double KO CAR T-cells exhibited increased stem cell-like TIM-3^−^TCF1^+^ CD8 T-cells (45) in the tumor and peripheral blood, although only *PRDM1/NR4A3* KO CAR T-cells showed a significant decrease in the frequencies of TIM-3^+^TCF1^−^ exhausted T-cells in the tumor (*P* < 0.05, Fig. 7F and G, fig. S7D).

To investigate whether *PRDM1/NR4A3* dual KO enhances CAR TIL effector function, we reactivated these TILs ex vivo and assessed intracellular cytokine production. Consistent with our in vitro results (Fig. 5D and E), *PRDM1* or *NR4A3* single KO failed to improve effector cytokine production, whereas *PRDM1/NR4A3* double KO CAR T-cells maintained higher polyfunctionality compared to control CAR T-cells (Fig. 7H, fig. S7E).

We also examined the antitumor activity of *PRDM1/NR4A3* KO CAR T-cells in the NALM-6 B-ALL model. Similar to the results obtained from the PC3-PSMA model, *NR4A3* single KO CD19 CAR T-cells failed to suppress tumor growth. *PRDM1* single KO moderately enhanced tumor control and survival (Fig. 7I to K). However, when *PRDM1* KO was combined with *NR4A3* KO, CAR T-cells induced rapid tumor clearance and durable antitumor efficacy (Fig 7I to K). Further, we assessed the efficacy of *PRDM1* and *NR4A3* single KO or *PRDM1/NR4A3* double KO mesothelin-directed CAR T-cells in an in vivo model of highly resistant pancreatic adenocarcinoma incorporating AsPC1 tumor cells expressing endogenous mesothelin (fig. S7F). In this study, only *PRDM1/NR4A3* double knockout anti-mesothelin CAR T-cells mediated a significant reduction in tumor burden over time (*P* < 0.05, fig. S7G).

Additionally, in association with robust antitumor activity against NALM-6 B-ALL (fig. S7H), we found that *PRDM1/NR4A3* KO enhanced CD19 CAR T-cell expansion compared to *AAVS1* KO CAR T-cells (fig. S7I). We also observed increased central memory T-cell differentiation and reduced proportions of peripheral blood CAR T-cells co-expressing multiple inhibitory receptors as compared to control CAR T-cells (fig. S7J to L). To evaluate the durability of *PRDM1/NR4A3* double KO CAR T-cell therapeutic efficacy, we conducted a study in which CAR T-cell-treated NSG mice were rechallenged with NALM-6 cells 40 days after the initial leukemia cell transfer. Because of the aggressive nature of this model, we administered a relatively high dose of CAR T-cells across all groups during the primary challenge to ensure initial tumor clearance. *PRDM1/NR4A3* dual KO CAR T-cells demonstrated better control of tumor growth than control CAR T-cells following rechallenge (Fig. 7L and M). These collective findings suggest that *PRDM1* depletion increases CAR T-cell expansion and mitigates dysfunction by increasing frequencies of TCF1^+^CD8^+^ T-cells and skewing fate away from the TIM-3^+^CD8^+^ state. *NR4A3* KO rescues the in vivo potency-enhancing effect of *PRDM1* KO on CAR T-cell function by reducing exhaustion and inducing durable effector activity.

## Discussion

The full therapeutic potential of CAR T-cell therapies in cancer is often hindered by poor expansion, persistence, and lack of durable effector function of the transferred cellular product. Unfortunately, to date, efficacy in solid tumors has been limited in particular (6–8), and the underpinnings of CAR T-cell intrinsic antitumor potency in these indications have not been elucidated. Our results suggest that cellular and molecular diversity of infused CAR T-cells is a major factor contributing to the variability in proliferative capacity and antitumor activity among patients with metastatic solid tumors. Quantifiable phenotypes associated with pre-infusion PSMA CAR T-cells in mCRPC also shared with CD19 CAR T-cell products in leukemia and lymphoma are potentially actionable by enriching desirable, or eliminating undesirable, cellular populations or functional states during ex vivo manufacturing. Further, our study identifies the mechanisms by which distinct CAR T-cell populations lead to therapeutic potency and offers an engineering strategy for potentiating enrichment of highly functional products to improve efficacy after infusion.

A central finding from our study is that the presence of stem cell-like *TCF7*-expressing CD8^+^ and absence of *HAVCR2*-expressing CD8^+^ exhausted T-cells are indicative of CAR T-cell expansion and antitumor activity in both non-hematopoietic and hematopoietic cancers, suggesting that the intrinsic fitness of the engineered T-cells, in addition to absolute T-cell number and spatial distribution within a patient’s tumor, is crucial for induction of effective tumor immunity. *TCF7*-expressing CD8^+^ T-cells are a distinct CD8^+^ T-cell population that expands and generates cytotoxic progenitors which are critical for elicitation of robust antitumor function in response to checkpoint blockade therapies (15, 45, 46). Additionally, the *TCF7* regulon not only associates with a favorable naïve T-cell state, but is also maintained in effectors among patients with long-term CAR T-cell persistence who experience durable remission (31). This is concordant with a previous report suggesting that *TCF7* mediates the persistence of CD8^+^ effector T-cells as well as their differentiation toward a central memory phenotype (47). Similar cell populations were identified in the CD4^+^ T-cell compartment, and CD4^+^ CAR T-cells may be critical for maintaining long-term persistence and antitumor function (48). Examination of larger patient cohorts is needed to validate and build on these molecular determinants of CAR T-cell activity and outcome.

In contrast to *TCF7*-expressing CD8^+^ T-cells, we found poorly functional cells expressing exhausted or dysfunctional transcriptomic programs associated with lack of in vivo PSMA CAR T-cell proliferative potency and PSA responses in mCRPC. *HAVCR2*-expressing CD8^+^ T-cells upregulated pathways associated with exhaustion and IFN responses, exhibited a lack of persistence, and resulted in poor CAR T-cell clinical efficacy; these cells also expressed a low degree of *TCF7*, which is predictive of negative outcome in patients treated with checkpoint inhibitors (49). Notably, we discovered that unlike *TCF7*-expressing CD8^+^ T-cells, *HAVCR2*-expressing CD8^+^ T-cells express a high abundance of *PRDM1* that has been reported to negatively regulate *TCF7* and mediate terminal T-cell differentiation and exhaustion (32, 33). We demonstrate that CRISPR/Cas9-mediated knockout of *PRDM1* not only successfully depletes *HAVCR2*-expressing CD8^+^ T-cell and increases *TCF7*-expressing CD8^+^ gene signatures during manufacturing, but also substantially enhances in vivo CAR T-cell proliferation and increased frequencies of TIM-3^−^TCF1^+^CD8^+^ peripheral blood and tumor-infiltrating CAR T-cells.

Although single deletion of *PRDM1* favorably modulates CAR T-cell differentiation, antitumor effector activity is ultimately hampered by this modification in the setting of chronic antigen exposure. *PRDM1* regulates CD8^+^ T-cell effector function and is required for granzyme B expression (32, 33). In acute LCMV infection, despite a reduction in cytotoxic molecule production, the cytolytic activity of virus-specific *PRDM1* KO CD8^+^ T-cells was marginally affected and both wild type and *PRDM1* KO CD8^+^ T-cells successfully cleared the infection (32). However, in chronic LCMV infection, the killing activity of LCMV-directed *PRDM1* KO CD8^+^ T-cells is impaired, suggesting that loss of *PRDM1* can profoundly compromise cytotoxicity in the context of exhaustion where the cytolytic potential of antigen-specific CD8^+^ T-cells is relatively low (33). Consistent with these previous studies, the cytotoxic capacity and effector cytokine production of *PRDM1* KO CAR T-cells were substantially compromised compared to conventional CAR T-cells after multiple rounds of tumor antigen exposure. In accordance with these findings, despite a profound increase in expansion capacity, *PRDM1* KO CAR T-cells minimally enhanced antitumor activity over control CAR T-cells in xenogeneic murine models of prostate cancer and aggressive B-cell leukemia, and ultimately became progressively dysfunctional due to exacerbated loss of effector activity. The marginally enhanced tumor control observed with this single modification may be attributed to the large increase in the number of *PRDM1* KO CAR T-cells in vivo, despite upregulation of exhaustion pathways and concomitantly diminished effector function.

Until now, *PRDM1* was presumed to induce exhaustion modules in antigen-specific T-cells (33). Detection of lower frequencies of peripheral blood *PRDM1* KO PSMA CAR T-cells, but not CAR TILs co-expressing multiple inhibitory receptors, suggests that reduction of inhibitory receptor expression in *PRDM1*-deficient CAR T-cells was insufficient to promote effective antitumor immunity. We therefore examined whether other transcription factors may regulate the exhaustion module and compensate for the absence of *PRDM1*. We discovered that *PRDM1* KO CAR T-cells chronically challenged with tumor targets display upregulation of a battery of exhaustion-associated transcription factors, including those belonging to the NR4A and TOX families which are reported to drive a cell-intrinsic program of T-cell hyporesponsiveness (50). These findings provide evidence that in the setting of *PRDM1* deletion, expression of certain inhibitory receptors and genes encoding exhaustion-related transcription factors are decoupled and downregulation of specific inhibitory receptors may instead be reflective of changes to the activation or differentiation state of CAR T-cells.

A recent study also analyzed *PRDM1* KO alone on antitumor T-cell responses (23). Interestingly, there are key differences between some of our findings and those presented in this report. While we build on the observation that *TCF7* is a critical determinant of CAR T-cell memory differentiation in the context of *PRDM1* deficiency using *TCF7* KO experiments, the progressive suppression of robust antitumor effector function mediated by *PRDM1* KO T-cells was not previously reported. Yoshikawa and colleagues (23) speculated that upregulation of TOX resulting from *PRDM1* deletion may diminish the antitumor response through upregulation of inhibitory receptors. Notably, we provide a functionally validated mechanism by which *PRDM1* KO regulates a battery of genes encoding exhaustion-related transcription factors, particularly NR4A3 not reported in (23), which together regulate CAR T-cell phenotype and effector activity. The decoupling of exhaustion-related transcription factor upregulation and inhibitory receptor expression observed in our study and not by Yoshikawa *et al*. may be attributed to several reasons. These discrepancies may include differences in the *PRDM1* KO CAR- and TCR-transgenic T-cells used and the transfer of a fewer number of engineered T-cells in our study. Nevertheless, our findings underscore the need to balance a reciprocal upregulation of NR4A3 and NFAT signaling in the setting of *PRDM1* deficiency to generate effective CAR T-cell-mediated responses in a variety of tumor types. Further, we provide a multiplex gene-editing strategy to potentiate the expansion of clinically relevant populations of stem-like CAR T-cells resistant to exhaustion.

T-cells in cancer and chronic viral infection differentiate into effector or exhausted T-cells, in association with global and site-specific changes to the epigenetic landscape, including transcription factor binding sites (51–53). Indeed, *PRDM1* KO increased accessibility of many exhaustion-related chromatin regions (such as *NR4A3, TOX*, and *TOX2*) that normally become accessible in CD8^+^ TILs (54); a number of these open regions colocalized with a BLIMP1 binding motif, suggesting that BLIMP1 may be acting as a transcriptional repressor. Further study is required to investigate whether BLIMP1 directly regulates chromatin accessibility through recruitment of epigenetic regulators such as G9a and Histone Deacetylase 2 (HDAC2) to form a functional complex regulating exhaustion-related transcription factor gene expression, as has been previously described (41). In addition to epigenetic regulation, *PRDM1* KO increased calcineurin-NFAT signaling, which is necessary and sufficient to induce exhaustion-related transcription factor expression (43, 44). This increased NFAT signaling may be attributed to prolonged exposure of *PRDM1* KO CAR T-cells to cancer cells in association with the delayed killing kinetics we demonstrated. Thus, our findings suggest that *PRDM1* can suppress expression of exhaustion-associated transcription factors by directly or indirectly regulating transcriptional and epigenetic reprogramming as well as the killing activity of CAR T-cells.

We recognize potential limitations of our present study. First, the tumors utilized in the mouse models are cell line-derived and may not behave exactly as primary tumors in humans. Further, use of immunocompromised mice engrafted with human cancer cell lines and T-cells falls short of recapitulating the interaction between gene-edited CAR T-cells and the native TME. Taking these limitations into account, the mouse models we used still offer good methods for assessment of CAR T-cell-augmenting regulators, though subsequent direct testing in human patients will be critical. Second, genotoxicity is a theoretical concern with dual KO of *PRDM1* and *NR4A3*. However, we recently reported results from a clinical trial where we edited three genes at separate loci with no evidence of genotoxicity (55). Finally, for clinical applications, it is important to recognize that if elimination of the *PRDM1/NR4A3* double-edited cells is compromised, a situation whereby a very large number of T-cells accumulate that are marginally less effective at tumor cell killing may considerably increase the risk of toxicity. This can be addressed in dose-escalation studies using a more conservative treatment regimen as compared to the initial doses evaluated in our prior phase I study (22). Given these uncertainties, initial clinical trials of this strategy should test *PRDM1/NR4A3* dual KO CAR T-cell products at comparably lower concentrations, prior to incorporation of lymphodepletion, which we anticipated to enhance T-cell proliferation, antitumor activity and treatment-related toxicity.

In summary, this study provides a framework for developing the next-generation of CAR T-cells by identifying cellular and molecular characteristics associated with clinical response and engineering CAR T-cells to favor enrichment of desirable cell populations, as well as depletion of undesirable cell populations, in infusion products. Our work also highlights the fundamental role of *PRDM1* in the regulation of human T-cell memory and provides a deeper understanding of how *PRDM1* mediates T-cell exhaustion (fig. S8). Additional experiments will be required to completely reveal the transcriptional networks involved. From a therapeutic standpoint, however, combined disruption of *PRDM1* and *NR4A3* expression or activity has the direct potential to generate genetically reprogrammed T-cells capable of augmenting both immediate and long-term antitumor responses, since this strategy potentiates the formation of long-lived memory cells resistant to exhaustion imposed by tumors.

## Materials and Methods

### Study design

The objective of this study was to define the functional effects of *PRDM1* and *NR4A3* KO in CAR T-cells. The number of biological replicates (referring to T cells from different healthy donors), type of statistical used and *P* values are reported in the figure legends. No statistical methods were used to pre-determine sample size. These were estimated based on preliminary experiments. All in vitro functional experiments in the main text were carried out at least three times and no outliers or other data points were excluded from our analyses. For animal experiments, NSG mice were treated with CAR T cells from one to two different healthy donors. Cages of mice were randomly assigned to treatment groups. Investigators were not blinded to group allocation during experiments and assessment of outcome.

Infusion product samples were acquired from patients with mCRPC who enrolled in a completed clinical trial of single-agent PSMA CAR T-cell therapy, which was approved by the Institutional Review Board (IRB) of the University of Pennsylvania. All ethical regulations were followed. This study is registered at ClinicalTrials.gov (NCT03089203) (22). Correlative analyses presented in this report are exploratory, given the post-hoc nature of the investigation. Peripheral blood mononuclear cells (PBMCs) were collected for small-scale CAR T-cell production by leukapheresis from healthy individuals. Study participants provided written informed consent according to the Declaration of Helsinki and the International Conference on Harmonization Guidelines for Good Clinical Practice. Correlative assays conducted on patient samples, such as quantitative polymerase chain reaction (qPCR) for CAR T-cell quantification, were carried out according to our previously published methods (20). Antitumor responses, as measured by maximum decline in PSA from baseline for the patients whose CAR T-cell infusion products were analyzed in this study, were previously reported (22).

### Cell lines

PC3, PSMA-expressing PC3, and NALM-6 cells engineered to express click beetle green luciferase and green fluorescent protein (CBG-GFP), were kindly provided by Carl H. June and Marco Ruella (University of Pennsylvania), respectively. AsPC1 cells were obtained from the American Type Culture Collection (ATCC). PC3 and PC3-PSMA cells were cultured in Dulbecco’s Modified Eagle Medium (DMEM) supplemented with 10% fetus bovine serum (FBS) and streptomycin/penicillin. NALM-6 and AsPC1 cells were maintained in Roswell Park Memorial Institute (RPMI)-1640 media supplemented with 10% FBS and streptomycin/penicillin (R10 media). HEK 293T cells, used for lentivirus production, were obtained from ATCC and cultured in R10 media. Low-passage banks of cells were tested for mycoplasma with a MycoAlert kit (Lonza), according to the manufacturer’s instructions. Authentication of cell lines was carried out by the University of Arizona Genetics Core, based on criteria established by the International Cell Line Authentication Committee. Short-tandem-repeat profiling revealed that these cell lines were above the 80% match threshold. Mycoplasma testing and authentication were routinely performed before and after molecular engineering.

### Lentivirus production

Vector construction and lentiviral production were conducted as previously described (56). In brief, CARs comprised of anti-PSMA (56), anti-CD19 (57), or anti-Mesothelin (58) single-chain variable fragments (scFv) fused to 4-1BB and CD3ζ stimulatory endodomains were subcloned into the pTRPE vector. Lentivirus supernatant was collected from 293T cells transfected with the pTRPE transfer vector and packaging plasmids using Lipofectamine 2000 (Thermo Fisher Scientific) and concentrated using ultracentrifugation.

### Lentiviral transduction and T-cell culture

Healthy donor T-cells were isolated from PBMCs using the Pan T Cell Isolation Kit according to the manufacturer’s instructions (Miltenyi Biotec). T-cells were activated with anti-CD3/CD28 antibody coated Dynabeads (Thermo Fisher Scientific) at 3:1 bead:cell ratio in T-cell media (OpTmizer CTS SFM media (Gibco) supplemented with 5% human AB serum and 100u/mL human IL-2). Following a 24-hour incubation, lentivirus encoding the PSMA CAR was added to the culture at a multiplicity of infection (MOI) of 2.5. CAR T-cell expansion was carried out as previously described (56).

### CRISPR/Cas9-mediated gene editing

Following T-cell activation as described above, beads were removed using a magnet on day 3 and electroporation was carried out with a P3 primary cell 4D-nucleofector kit (Lonza). 2 × 10^6^ CAR T-cells were transfected with 12 μg TrueCut S. pyogenes Cas9 (Invitrogen) and 0.2nmol of chemically-modified tracrRNA and crRNA (Integrated DNA Technologies). Following electroporation, CAR T-cells were cultured in T-cell media. The crRNA sequences used in this study were: *AAVS1*: 5’-CCATCGTAAGCAAACCTTAG-3’, *PRDM1*: 5’-CATCAGCACCAGAATCCCAG-3’, *TCF7*: 5’-TCAGGGAGTAGAAGCCAGAG-3’, *NR4A3*: 5’-CCTTGGCAGCACTGAGATCA-3’. The frequency of targeted mutations generated by double strand break were determined by Sanger sequencing and subsequent TIDE (tracking of indels by decomposition) analysis. Primers used for targeted amplification were: *PRDM1*-F1: tctcagaaggagccacaggaacgg, *PRDM1*-R1: cacccaccctatgctgcaagttgc, *NR4A3*-F1: gaggagaggatgacacttcctctctgtttc, *NR4A3*-R1: ctgcccagcacctccatgtacttcaagcag. Western blot and flow cytometric analysis were conducted to confirm KO at the protein level.

### Western blot analysis

T-cells (1 × 10^6^ cells) were suspended in a low-salt lysis buffer (10 mM HEPES, pH 7.9, 10 mM KCl, 0.1 mM EDTA, 0.1 mM EGTA, 1 mM DTT, 0.5 mM PMSF, 2 μg/ml aprotinin, 2 μg/ml leupeptin) and allowed to swell on ice for 30 minutes. After centrifugation (1000 × g), supernatants obtained from cells lysates (30 μg) were analyzed by 10% SDS-polyacrylamide gel electrophoresis and electrophoretically transferred to PVDF membranes (Millipore). The membranes were washed with phosphate-buffered saline (PBS) containing 0.1% Tween 20 (PBST), and then blocked for 1-hour in 5% skim milk in PBST. After washing with PBST, membranes were incubated overnight with one of the following antibodies: Mouse monoclonal anti-β-Tubulin (Sigma-Aldrich #T8318; 1:100) or rabbit monoclonal anti-BLIMP1 (Cell Signaling #9115; 1:100) or mouse monoclonal anti-NR4A3 (Sigma-Aldrich #SAB1404566; 1:100). Membranes were washed with PBST and treated with 1:1000 diluted horseradish peroxidase-coupled goat anti-mouse or anti-rabbit secondary antibodies (Thermo Fisher Scientific) in PBST for 1-hour. After washing, the membranes were incubated in Pierce ECL western blotting substrate (Thermo Fisher Scientific) and visualized on X-ray film.

### Flow cytometry

Cell surface anti-human antibodies were diluted in FACS buffer (PBS + 2% FBS). PSMA CAR expression was measured using an allophycocyanin (APC)-conjugated recombinant human PSMA protein (Sino Biological). T-cell immunophenotyping was carried out using the following antibodies: PD-1-Brilliant Violet (BV) 421 (BioLegend #329920; 1 μL/100 μL), CD45-BV570 (BioLegend #304226; 1 μL/100 μL), CD8-BV650 (BioLegend #301042; 1 μL/100 μL), CD8-APC-H7 (BD Biosciences #560179; 1 μL/100 μL), CD4-BV785 (BioLegend #317442; 1 μL/100 μL), TIM-3-phycoerythrin (PE) (BioLegend #345006; 1 μL/100 μL), CCR7-PE-CF594 (BD Biosciences #562381; 1 μL/100 μL), CD62L-PE-Cy5 (BioLegend #304808; 1 μL/100 μL), LAG3-PE-Cy7(eBioscience #25-2239-42; 1 μL/100 μL), hCD45-APC (BD Biosciences #340943; 1 μL/100 μL), murine CD45-Peridinin chlorophyll protein (PerCP)-Cy5.5 (BioLegend #103132; 1 μL/100 μL), CD127-BV570 (BioLegend #351307; 1 μL/100 μL), human leukocyte antigen (HLA)-DR-Alexa Fluor 700 (BioLegend #307625; 1 μL/100 μL), CD25-APC (eBioscience #17-0259-42; 1 μL/100 μL). For intracellular staining, cells were first permeabilized and washed using the FoxP3 Transcription Factor Staining Buffer Kit (eBioscience) and subsequently stained with following antibodies: IL2-PE-CF594 (BD Biosciences #562384; 3 μL/100 μL), IFN-γ-BV570 (BioLegend #502534; 3 μL/100 μL), TNF-α-Alexa Fluor700 (BioLegend #502928; 3 μL/100 μL), Perforin-BV421 (BioLegend # 353307; 2 μL/100 μL), Perforin-APC (BioLegend #308112; 2 μL/100 μL), Granzyme B-PE-Cy5.5 (Invitrogen #GRB18; 0.04 μL/100 μL), TCF1-Alexa Fluor 488 (Cell Signaling Technology #6444S; 1 μL/100 μL), TOX-APC (Miltenyi Biotech #130-107-785; 3 μL/100 μL), NFATC1-PE (BioLegend #649606; 3 μL/100 μL) according to our previously published methods (20). Samples were then analyzed using an LSRFortessa (BD Biosciences), FlowJo software (FlowJo, LLC) or FCS Express (De Novo Software).

### CAR T-cell restimulation assay

CAR T-cell expansion capacity and effector function were assessed using a restimulation assay, as previously described (20, 56, 59). Briefly, *AAVS1* KO, *PRDM1* KO, *TCF7* KO, *NR4A3* KO, *PRDM1* + *TCF7* dKO, *PRDM1* + *NR4A3* dKO PSMA CAR-positive T-cells were isolated using a Biotin-goat anti-mouse IgG F(ab)2 fragment (Jackson ImmunoResearch #115-065-072) and anti-biotin kits (Miltenyi Biotech). Following isolation, CAR T-cell purity was confirmed by flow cytometric analysis and CAR T-cells were then serially exposed (every 2 to 5 days) to irradiated PC3-PSMA cells at an effector-to-target (E:T) ratio of 3:1 or 1:1. Restimulation assays were carried out for 25 to 30 days, since cell counts become largely unreliable beyond the fifth and sixth rounds of stimulation due to senescent proliferative arrest and a marked decrease in the viability of chronically stimulated CAR T-cells. Supernatants were collected 24-hours post-tumor challenge for cytokine analysis using the LEGENDplex human CD8 panel (BioLegend), and absolute numbers of T-cells in culture were monitored using a Luna automated cell counter (Logos Biosystems) during the assay.

### Cytotoxicity assay

The longitudinal killing capacity of engineered CAR T-cells against PC3-PSMA cells was assessed using the xCELLigence system (ACEA Biosciences Inc.). CAR-expressing T-cells were magnetically enriched prior to the cytotoxicity assay. 2 × 10^4^ PC3-PSMA cells were seeded in E-Plate VIEW 96 PET microwell plates. After 24-hours, PSMA CAR T-cells or control (untransduced) T-cells were added to wells containing tumor targets to achieve the desired E:T ratios. 20% Tween 20 was added to separate wells consisting of PC3-PSMA cells alone as a full lysis control. Electrical impedance was monitored in 20-minute intervals over 7 days and cytotoxicity was assessed by calculating the normalized cell index and % cytolysis.

### Quantitative real-time PCR (qRT-PCR)

After 2 to 4 rounds of restimulation, CD8^+^ CAR T-cells were isolated from culture using CD8 microbeads (Miltenyi Biotec) for qRT-PCR. First, total RNA was extracted from CD8^+^ CAR T-cells using RNA Clean & Concentrator kits (Zymo Research). cDNA was then synthesized using the PrimeScript 1^st^ strand cDNA Synthesis Kit (Takara Bio) per the manufacturer’s protocol and qRT-PCR was conducted using Applied Biosystems TaqMan Fast Advanced Master Mix (Thermo Fisher Scientific) on the QuantStudio3 (Applied Biosystems). The primer/probe sets used for these experiments were as follows: CD3E: Hs01062241_m1, NR4A2: Hs00428691_m1 and NR4A3: Hs00545009_g1 (Thermo Fisher Scientific).

### Mouse xenograft studies

Mouse studies were performed with 6- to 8-week-old male NOD/SCID/IL-2Rγ-null (NSG) mice in compliance with a University of Pennsylvania Institutional Animal Care and Use Committee approved protocol. For the subcutaneous PC3-PSMA model, 10^6^ or 5 × 10^6^ PC3-PSMA-CBG-GFP tumor cells were premixed with Matrigel (Corning) and injected into the flanks of NSG mice. When the average tumor size reached 150 to 200 mm^3^ or 500 mm^3^, 3.5 × 10^5^ PSMA CAR T-cells were injected intravenously. Tumor growth weekly monitored weekly by taking caliper measurements (tumor volume = (length × width^2^)/2). Animals were euthanized when tumor volume exceeded 1500 mm^3^ or 2 cm in diameter. For the intraosseous PC3-PSMA model, male NSG mice were intrafemorally transplanted with 2 × 10^5^ PC3-PSMA cells. On day 27, 1 to 2 × 10^5^ PSMA CAR T-cells were infused intravenously, and tumor burden was measured by injecting mice intraperitoneally with luciferin and quantifying bioluminescence using an IVIS Spectrum In Vivo Imaging System (PerkinElmer). To characterize CAR T-cell phenotype and function, peripheral blood and tumor tissues were isolated from mice. Blood samples were obtained at peak CAR T-cell expansion by cheek bleeding. Tumors isolated on day 13 post-CAR T-cell injection were minced with a scalpel and treated with 100 U/mL collagenase IV and 0.25mg/mL DNase I for 1 hour at 37°C. The absolute number of human T-cells in the peripheral blood and tumors was quantified using 123count eBeads (Thermo Fisher Scientific). To assess the effector function of TILs, these ex vivo isolated T-cells were stimulated with 50 ng/mL phorbol 12-myristate 13-acetate (PMA) and 1mg/mL ionomycin in the presence of 5 ug/mL Brefeldin A for 6-hours and expression of IL-2, IFN-γ and TNF-α were assessed by intracellular staining. We used PMA/ionomycin stimulation to assess TIL effector function to eliminate potential confounding variables associated with antigen receptor expression or signaling.

To examine the antitumor efficacy of CAR T-cells in pancreatic cancer, 4 × 10^6^ AsPC1 cells were premixed with Matrigel and subcutaneously injected into NSG mice. On day 30, when tumor volume reached 300 to 400 mm^3^, mesothelin-directed CAR T-cells were intravenously injected, and tumor growth was monitored using caliper measurements. For the B-cell acute lymphoblastic leukemia model, NSG mice were intravenously injected with 1 × 10^6^ NALM-6-CBG cells. On day 7 post-tumor injection, 3 × 10^5^ CD19 CAR T-cells were intravenously infused, and tumor growth was assessed twice weekly using bioluminescent imaging (20). Peripheral blood was isolated on day 24 and flow cytometric T-cell immunophenotyping was carried out.

To assess the memory recall response of CAR T-cells, NSG mice were engrafted with 10^5^ NALM-6-CBG cells. On day 6 post-tumor injection, 2 × 10^6^ CD19 CAR T-cells were intravenously infused (*n* = 9 to 10 mice per group). The mice that had exhibited initial control of NALM-6 cells and survived were rechallenged with 2 × 10^6^ NALM-6 cells on day 40. Tumor growth was monitored using bioluminescence imaging, as described above.

### Single-cell RNA-seq

PSMA CAR T-cell infusion products from 5 different patients were thawed in a 37°C water bath and dead cells were eliminated using the Dead Cell Removal Kit (Miltenyi Biotec). PSMA CAR-positive T-cells were enriched by magnetic cell separation using a Biotin-goat anti-mouse IgG F(ab)2 fragment (Jackson ImmunoResearch #115-065-072) and anti-biotin kits (Miltenyi Biotech). scRNA-seq libraries were prepared using Chromium Single Cell 3’ v3.1 Reagent Kits (10X Genomics) following the manufacturer’s instructions. Isolated CAR T-cells were washed and resuspended in PBS containing 0.04% BSA and about 20,000 cells were loaded per reaction to capture about 10,000 cells. Sequencing was performed on a Novaseq 6000 (Illumina) at a depth of at least 20,000 reads per cell.

Reads were aligned to the human reference genome (GRCh38) using Cell Ranger version 6.0.0. Subsequent quality control and downstream analyses were performed using Seurat 4.0. Cells were filtered based on the following criteria to eliminate low-quality cells: 1) a minimum of 1000 genes and a maximum of 6000 genes detected per cell; and 2) less than 15% of mitochondrial gene counts. After quality control, 20,702 cells remained. Gene-cell matrices from five CAR T-cell products were then integrated using SelectIntegrationFeatures, PrepSCTIntegration, FindIntegrationAnchors, and IntegrateData functions in Seurat. CD4-positive and CD8-positive cells were clustered using FindNeighbors/FindClusters, followed by differential gene expression analysis using the FindClusters command. Module scores were then calculated using AddModuleScore to assess the enrichment of gene signatures in CD4 and CD8 subclusters.

### Bulk RNA-seq

On days 0, 5 and 20 of the CAR T-cell restimulation assay, before stimulation and after the first and fourth tumor challenge respectively, CD8^+^ CAR T-cells were isolated using CD8 microbeads (Miltenyi Biotec) and total mRNA was extracted with RNA Clean & Concentrator kits (Zymo Research). Bulk RNA-seq was conducted by Novogene using the Novaseq6000 system (paired-end 150bp) at 40 × 10^6^ reads per sample. Reads were pseudoaligned to the human genome (GRCh38) transcriptomes using kallisto v0.46.0.

Differential expression analyses between *AAVS1* KO PSMA CAR T-cells and *PRDM1* KO PSMA CAR T-cells were performed using the edgeR v3.34.0 and limma v3.48.0 packages. Briefly, expression data were normalized using a trimmed mean of log expression ratios method and transformed into log2(counts per million). Linear models were used to assess differential expression, and *P* values were adjusted using the Benjamini-Hochberg method. Gene set enrichment analysis was conducted using GSEABase v1.54.0, clusterProfiler v4.0.2, and msigdbr v7.4.1.

### ATAC-seq analysis

After CAR T-cell manufacturing, dead cells were eliminated using the Dead Cell Removal Kit (Miltenyi Biotec) and CD8^+^ CAR T-cells were isolated using CD8 microbeads (Miltenyi Biotec). 100,000 cells per sample were cryopreserved. Nuclei were isolated from CD8^+^ T-cells for each replicate, followed by the transposition reaction in the presence of Tn5 transposase (Illumina, Inc.) for 45 minutes at 37°C. Purification of transposed DNA was then completed with the MinElute Kit (Qiagen) and fragments were barcoded with dual indexes (Illumina Nextra). Library preparation and sequencing were performed by Novogene using NovaSeq 6000 (paired-end 150bp reads) at a depth of 30 × 10^6^ reads per sample.

FASTQ files for each sample were trimmed of adapter contamination using cutadapt (https://github.com/marcelm/cutadapt/). They were aligned to the hg19 reference genome using Bowtie2, restricting to properly aligned and properly paired reads between 10 and 1000 base pairs. Mitochondrial reads were removed (https://github.com/jsh58/harvard/blob/master/removeChrom.py). Files were sorted using samtools, and PCR duplicates were removed using Picard. BAM files were indexed using samtools in order to visualize tracks in IGV. Peak calling was performed with MACS2 with a false discovery rate (FDR) q-value of 0.01. The R package Diffbind was used to remove ENCODE blacklisted regions (https://sites.google.com/site/anshulkundaje/projects/blacklists), then to identify peaks differentially opened between the control and the *PRDM1* knockout. The findMotifsGenome script from HOMER was used to map the hg19 genome for occurrences of the BLIMP1 motif (derived from ENCODE data accessible by GEO at GSE31477) and the NFAT2 motif (60).

### Statistical analyses

Raw, individual-level data are presented in data file S1. Normality analyses were conducted using Shapiro-Wilk test and D’Agostino & Pearson tests. Pairwise comparisons were performed using the Mann Whitney U test and Student’s t-test as appropriate. For comparisons of three or more groups, one-way ANOVA with Tukey’s multiple comparisons test and Kruskal-Wallis test with a post-hoc Dunn’s multiple comparison test were used. Mouse survival was assessed using the Gehan-Breslow-Wilcoxon test. Statistical tests were performed in Prism 9 (GraphPad Software) and *P* values <0.05 were considered significant.

## Supplementary Material

Supplementary Material

Data file S1. Raw, individual-level data

## Data Availability

All data associated with this study are in the paper or supplementary materials. Raw and analyzed sequencing datasets have been deposited to Gene Expression Omnibus (GEO) under the accession number GSE210265. Additional requests for raw and analyzed data or materials should be made to the PENN Center for Innovation (pciinfo@pci.upenn.edu) and will be promptly reviewed to determine whether the application is subject to any intellectual property or confidentiality requirements. Patient-related information not included in this report were collected as part of a clinical trial and might be subject to patient confidentiality. Any data and materials that can be shared will be released following execution of a material transfer agreement.
