# Supplementary material for: BLIMP1 and NR4A3 Transcription Factors Reciprocally Regulate Antitumor CAR T-cell Stemness and Exhaustion
Source: Sci Transl Med. Author manuscript; Available in PMC 2023 Jun 10. (PMC10257143; doi:10.1126/scitranslmed.abn7336)
Supplement: Supplementary Material [file NIHMS1902477-supplement-Supplementary_Material.docx]

**Supplementary Material**

**BLIMP1 and NR4A3 Transcription Factors Reciprocally Regulate Antitumor CAR T-cell Stemness and Exhaustion**

In-Young Jung^1,2,3,4^, Vivek Narayan^3,5^, Sierra McDonald^6,7,8,9^, Andrew J. Rech^2,4,6^, Robert Bartoszek^1,2,3,4^, Gwanui Hong^10^, Megan M. Davis^2,4^, Jun Xu^2,4^, Alina C. Boesteanu^2^, Julie S. Barber-Rotenberg^2^, Gabriela Plesa^2^, Simon F. Lacey^2,4^, Julie K. Jadlowsky^2^, Donald L. Siegel^2,4^, Dana M. Hammill^2^, Park F. Cho-Park^10^, Shelley L. Berger^6,7,8,9^, Naomi B. Haas^3,5^, and Joseph A. Fraietta^1,2,3,4†^

*^1^Department of Microbiology, Perelman School of Medicine, University of Pennsylvania, Philadelphia, PA (19104)*

*^2^Center for Cellular Immunotherapies, Perelman School of Medicine, University of Pennsylvania, Philadelphia, PA (19104)*

*^3^Abramson Cancer Center, Perelman School of Medicine, University of Pennsylvania, Philadelphia, PA (19104)*

*^4^Department of Pathology and Laboratory Medicine, Perelman School of Medicine, University of Pennsylvania,*

*Philadelphia, PA (19104)*

*^5^Department of Medicine, Perelman School of Medicine, University of Pennsylvania, Philadelphia, PA (19104)*

*^6^Parker Institute for Cancer Immunotherapy, University of Pennsylvania, Philadelphia, PA (19104)*

*^7^Epigenetics Institute, Perelman School of Medicine, University of Pennsylvania, Philadelphia, PA (19104)*

*^8^Department of Cell and Developmental Biology, Perelman School of Medicine, University of Pennsylvania,*

*Philadelphia, PA (19104)*

*^9^Department of Genetics, Perelman School of Medicine, University of Pennsylvania, Philadelphia, PA (19104)*

*^10^Department of Systems Pharmacology & Translational Therapeutics, Perelman School of Medicine, University of*

*Pennsylvania, Philadelphia, PA (19104)*

^†^Corresponding author: [jfrai@upenn.edu](mailto:jfrai@upenn.edu)


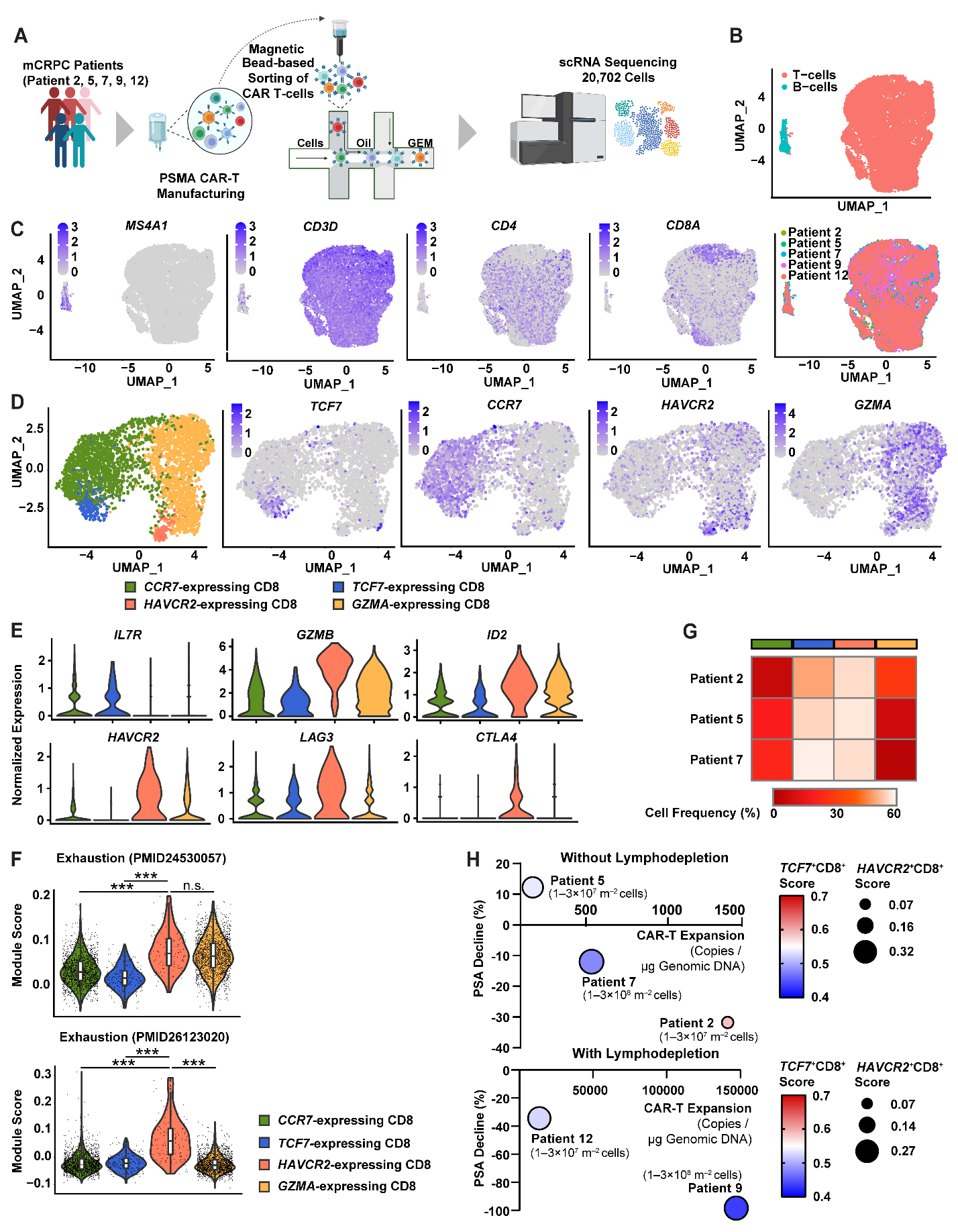


**Figure S1. Single-cell RNA-seq study design and subsequent analysis.** (**A**) A schematic of study design and sample processing for single cell RNA-sequencing (scRNA-seq) analysis of metastatic castration-resistant prostate cancer (mCRPC) patient prostate-specific membrane antigen (PSMA) chimeric antigen receptor (CAR) T-cell infusion products. (**B**) Infusion products were analyzed by scRNA-seq using integrated data from five patients (total 20,702 cells that passed quality control). Clusters are labeled with cell types (top) and patient origin (bottom). (**C**) The uniform manifold approximation and projection (UMAP) plot displays mRNA transcripts for selected genes (*MS4A1*, *CD3D*, *CD4*, and *CD8A*) expressed in CAR T-cell infusion products. (**D**) Expression of CD8 T-cell cluster-defining markers is shown. (**E**) Violin plots show expression of markers associated with CD8^+^ T-cell early memory differentiation, cytotoxicity, and exhaustion. (**F**) Scores of gene signatures associated with T-cell exhaustion are shown for the indicated CD8 T-cell populations (PMID24530057, PMID26123020). ****P* < 0.001, n.s.: not significant, as measured by a Kruskal-Wallis test with a post-hoc Dunn’s multiple comparison test. (**G**) Frequencies of *CCR7*-, *TCF7*-, *HAVCR2*- and GZMA-expressing CD8^+^ clusters in each CAR T-cell infusion product are shown. (**H**) Bubble plots show the associations between mCRPC patient CAR T-cell infusion product *TCF7*- and *HAVCR2*-expressing CD8^+^ T-cell gene signature scores, prostate-specific antigen (PSA) response and peripheral blood (PB) CAR T-cell expansion. CAR T-cell doses and lymphodepletion conditions for each patient are indicated.

**
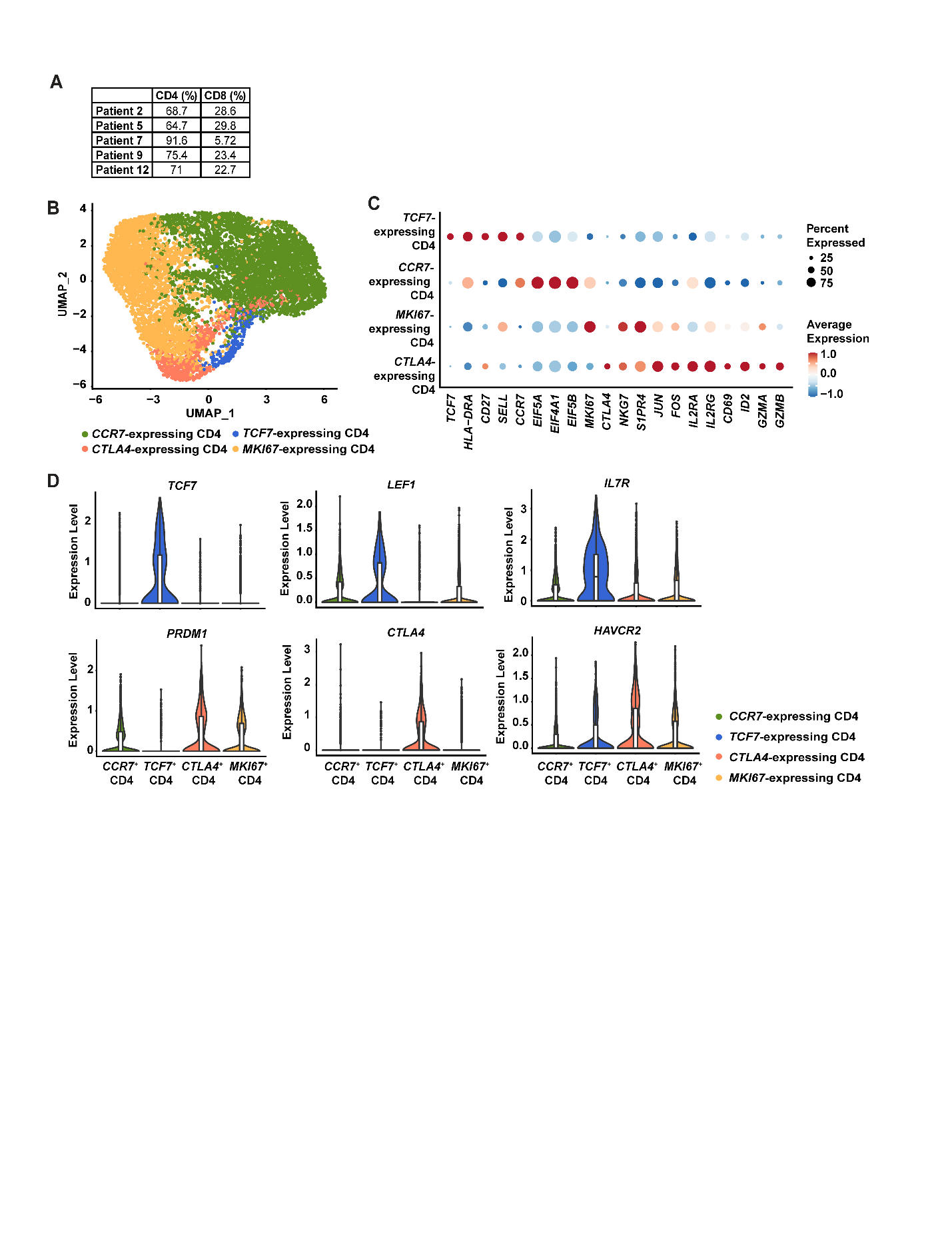
**

**Figure S2. Single-cell RNA-seq analysis of infusion product CD4^+^ T-cells.** (**A**) Frequencies of CD4^+^ and CD8^+^ T-cells in mCRPC patient CAR T-cell infusion products are shown. (**B**) The UMAP plot displays sub-clustering of infusion product CD4**^+^** T-cells. (**C and D**) Cluster-defining marker gene expression profiles of CD4^+^ subclusters.

**
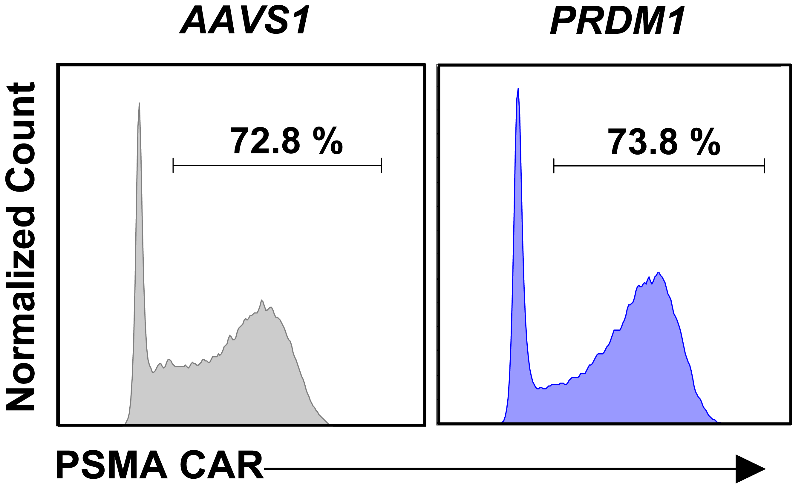
**

**Figure S3. PSMA CAR expression at the end of gene-edited CAR T-cell manufacturing.** Flow cytometry histograms are shown depicting PSMA CAR expression in *PRDM1* knockout (KO) compared to *AAVS1* KO CAR T-cells (representative CAR T-cell data from *n* = 3 different donors).

**
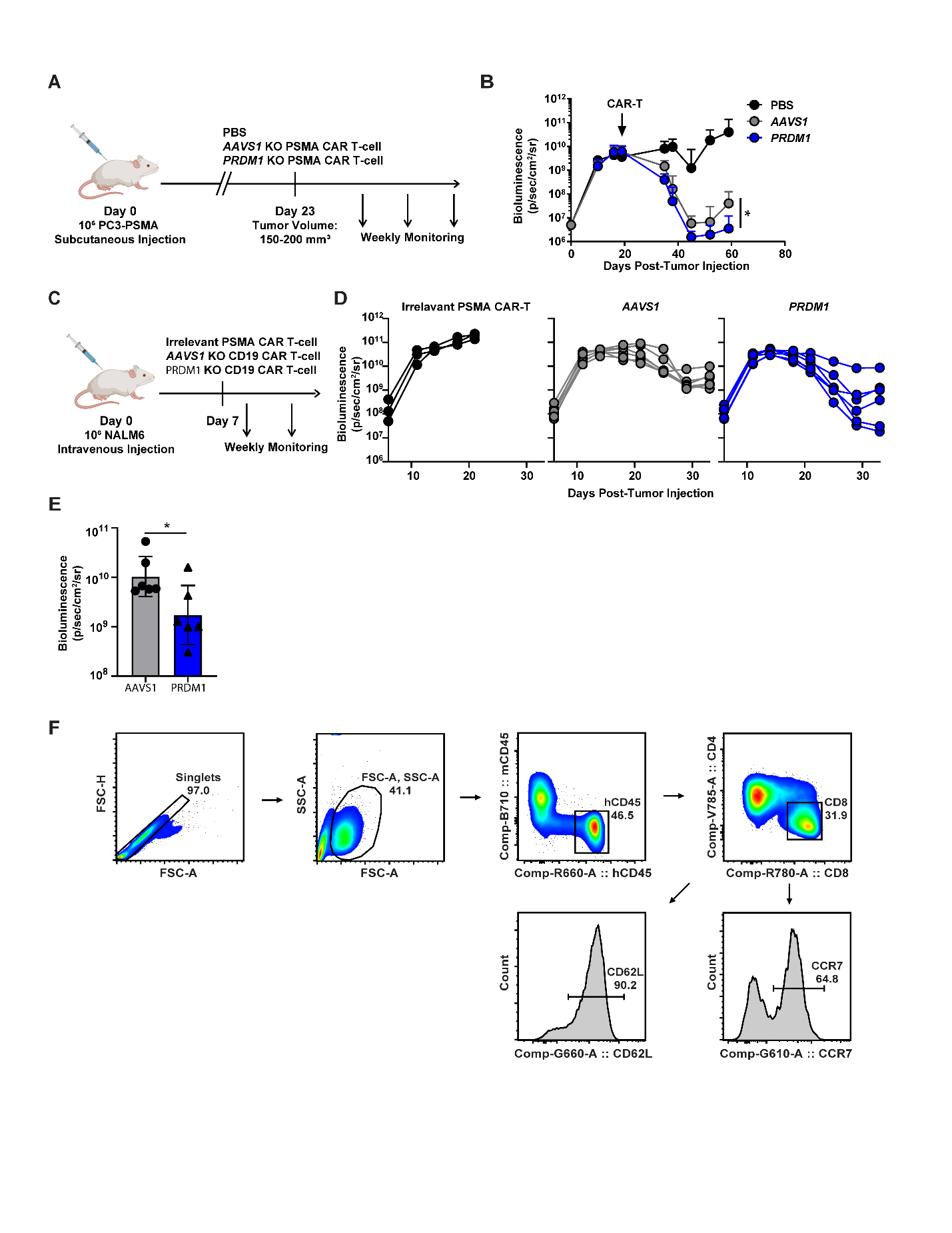
**

**Figure S4. In vivo studies using prostate cancer and B-cell acute lymphoblastic leukemia (B-ALL) xenogeneic mouse models to examine the antitumor potency of *PRDM1* KO CAR T-cells.** (**A**) A schematic of the low tumor burden PC3-PSMA xenograft mouse model is shown. (**B**) Tumor burden was monitored by bioluminescent imaging. Data depict geometric mean ± S.D.; *n* = 6, representative of two independent experiments. (**C**) A schematic of the NALM-6 xenograft model. Briefly, NOD/SCID/IL-2Rγ-null (NSG) mice were intravenously injected with 1 × 10^6^ NALM6-CBG cells. 1 × 10^5^ CD19 CAR T-cells and negative control, PSMA CAR T-cells, were administered 7 days post-tumor injection (*n* = 6) (**D**) NALM-6 B-ALL growth was monitored by bioluminescent imaging. (**E**) Tumor burden on day 18 post-CAR T-cell injection is shown. Data indicate the geometric mean ± S.D.; *n* = 6. (**F**) Representative flow cytometry plots show a gating strategy to characterize the immunophenotype of CAR T-cells in the peripheral blood. Data in (**B**) and (**E**) were analyzed by Mann Whitney U tests. **P* < 0.05. FSC-A, forward scatter area; FSC-H, forward scatter height; SSC-A, side scatter area.


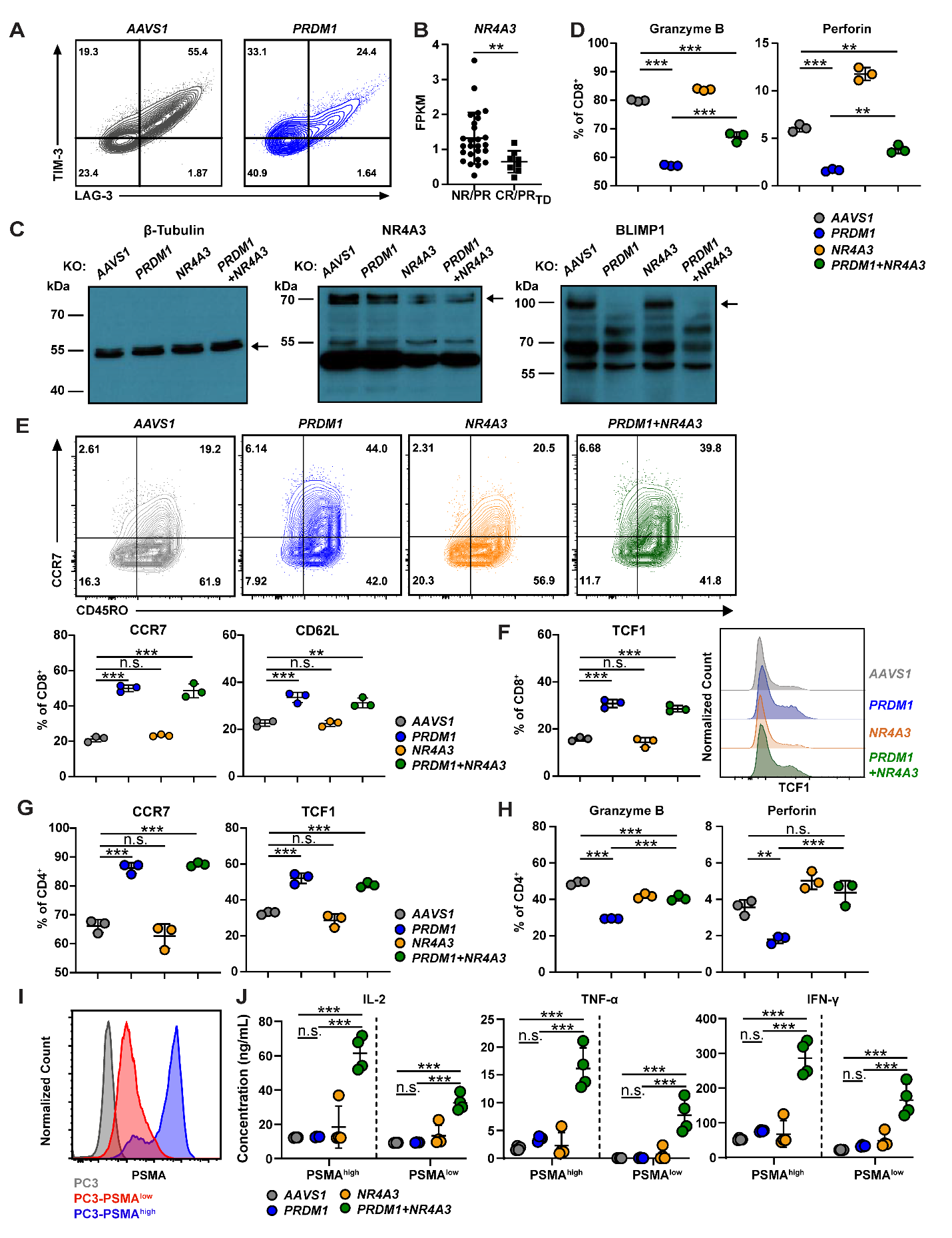


**Figure S5. Early memory differentiation phenotypes and cytotoxic profiles of *PRDM1*/*NR4A3* dual KO CAR T-cells in vitro.** (**A**) Flow cytometry contour plots showing frequencies of gene-edited CAR T-cells expressing TIM-3 and LAG-3 inhibitory receptors. (**B**) Comparison of *NR4A3* expression in CD19 CAR T-cell infusion products from patients with CLL (CR: complete response; PR_TD_: very good partial response; PR: partial response; no response). FPKM, Fragments per kilo base of transcript per million mapped fragments. (**C**) Representative Western blots showing BLIMP1 and NR4A3 expression in gene-edited CAR T-cells. (**D**) Granzyme B and Perforin expression was measured in CD8^+^ CAR T-cells after five rounds of restimulation with PC3-PSMA tumor targets. (**E**) Expression of early memory T-cell markers (CCR7 and CD62L) on gene-edited CD8^+^ CAR T-cells at 5 days post-tumor challenge is shown. (**F**) Flow cytometry analysis of TCF1 expression is shown at 5 days post-PC3-PSMA tumor stimulation. (**G**) Frequencies of CCR7- and TCF1-expressing CD4^+^ CAR T-cells are shown in samples collected 5 days post-tumor challenge. (**H**) Granzyme B and Perforin expression in CD4^+^ CAR T-cells after the fifth tumor restimulation are shown. (**I**) PSMA expression of PC3 cell lines is shown. (**J**) CAR T-cells were repetitively challenged with PC3-PSMA^high^ cell lines. After the fifth tumor challenge, PSMA CAR T-cells were isolated and co-cultured with PC3-PSMA^high^ or PC3-PSMA^low^ cell lines. Effector cytokines were measured 24 hours following co-culture. Data depict mean ± S.D. (*n* = 3). **P* < 0.05, ***P* < 0.01, ****P* < 0.001, n.s., not significant. Data were analyzed by a one-way ANOVA test with a post-hoc Tukey’s multiple comparison test .


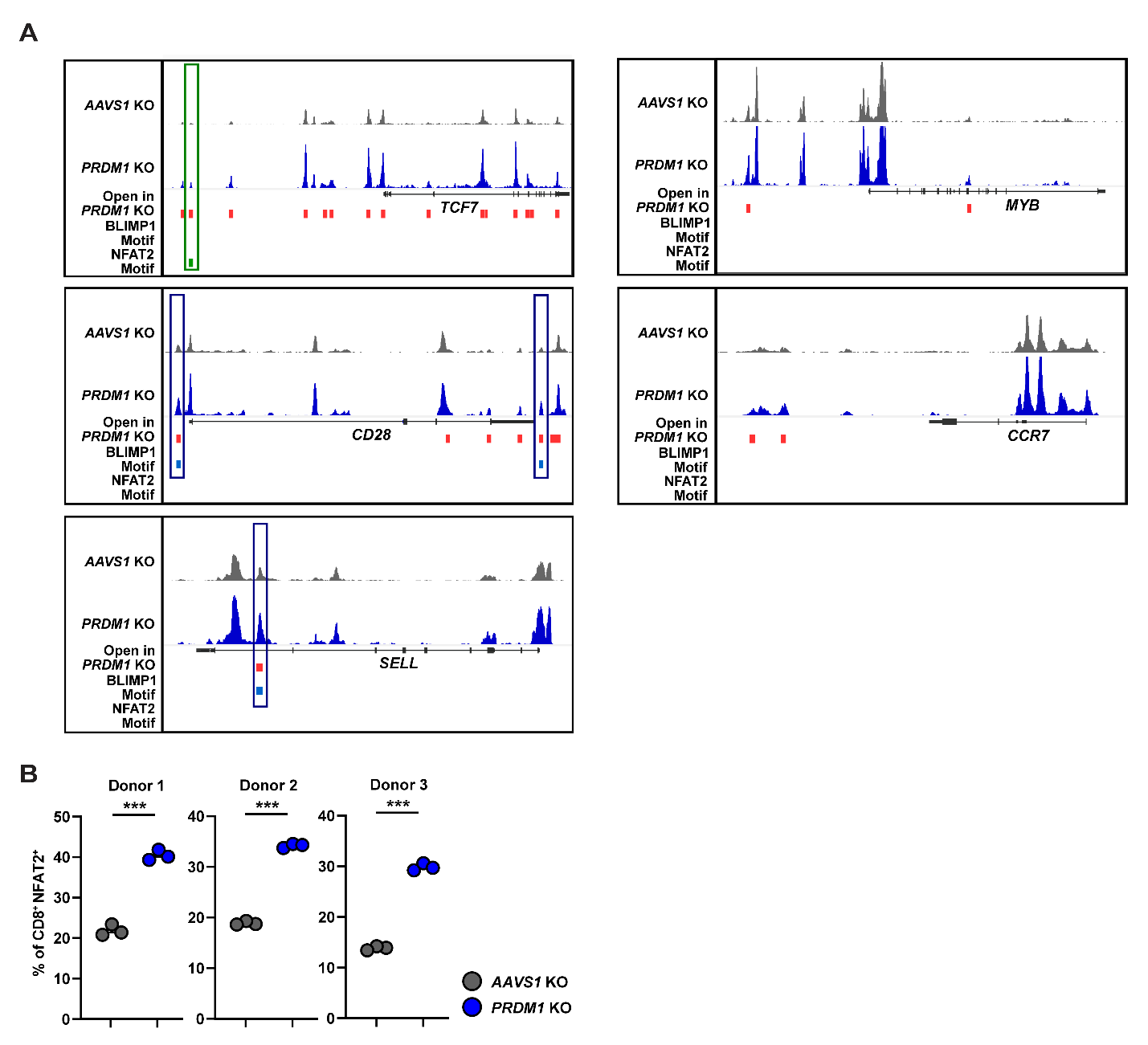


**Figure S6. *PRDM1* KO increases chromatin accessibility of T-cell memory-related genes and NFAT2 expression.** (**A**) Assay for Transposase-Accessible Chromatin with sequencing (ATAC–seq) tracks of memory-related gene loci in *AAVS1* and *PRDM1* KO CAR T-cells are shown. Opened chromatin regions in *PRDM1* KO CAR T-cells and binding motifs of BLIMP1 and NFAT2 are shown. (**B**) Frequencies of NFAT2-expressing CD8^+^ CAR T-cells were measured by flow cytometry. *n* = 3; two-tailed *t*-test.


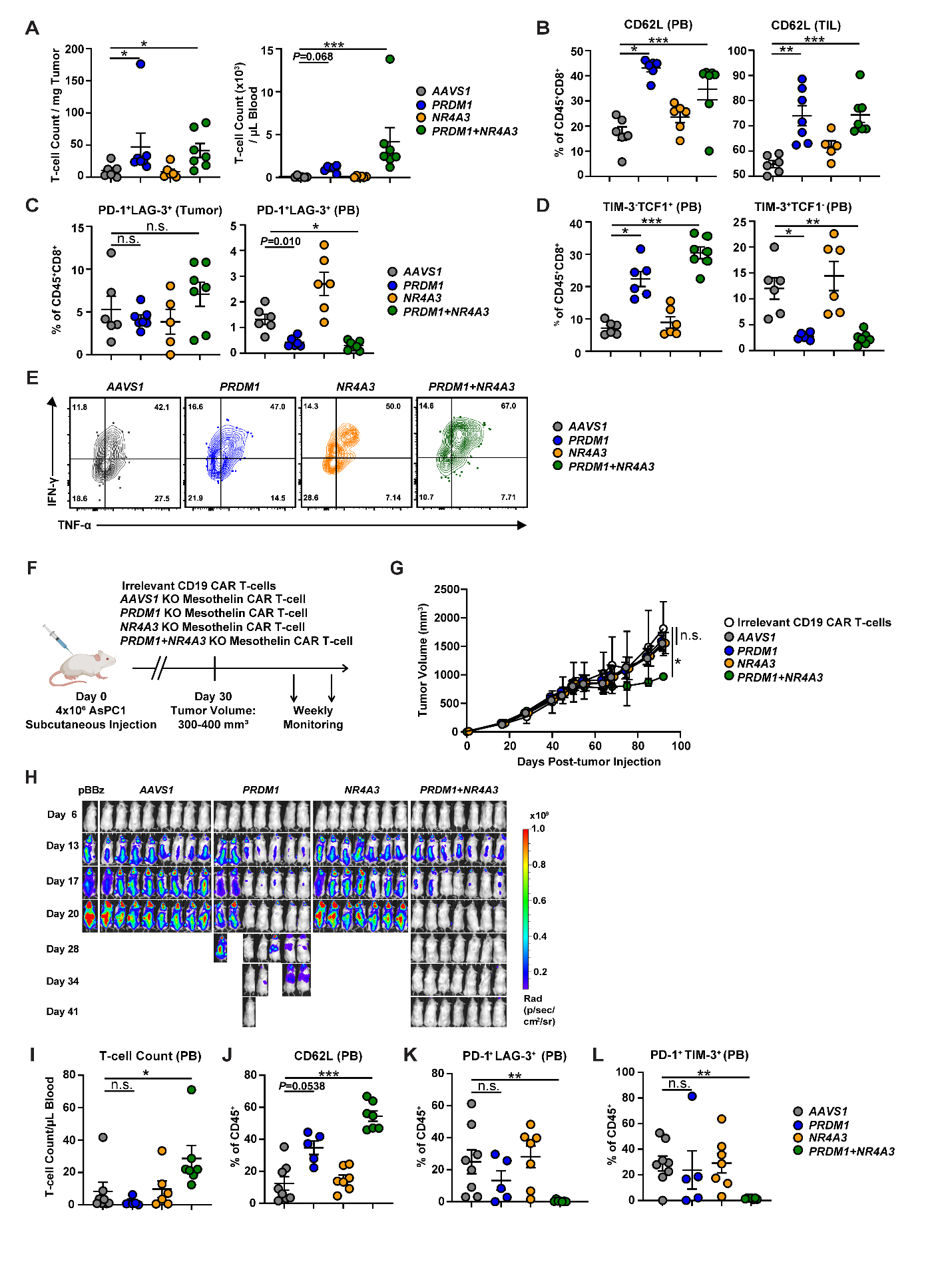


**Figure S7. *PRDM1*/*NR4A3* dual KO enhances CAR T-cell antitumor efficacy in xenograft mouse models of adoptive cell immunotherapy.** (**A to E**) *AAVS1* KO, *PRDM1* KO, *NR4A3* KO, and *PRDM1*/*NR4A3* dual KO PSMA CAR T-cells were isolated from subcutaneous PC3-PSMA tumors and immunophenotyped by flow cytometry. (**A**) Absolute numbers of human CD45^+^ (hCD45) T-cells in tumors (left) and the peripheral blood (PB; right) are shown. Frequencies of gene-edited CAR T-cells isolated from the peripheral blood or tumors expressing (**B**) CD62L and (**C**) PD-1 as well as LAG-3. TIL, tumor-infiltrating lymphocyte. (**D**) Evaluation of the proportions of CAR T-cells expressing TIM-3 and TCF1 in the peripheral blood of tumor-bearing mice. (**E**) CAR T-cells infiltrating the tumor were reactivated with phorbol 12-myristate 13-acetate (PMA) and ionomycin for 6 hours, followed by intracellular staining for interferon (IFN)-γ, tumor necrosis factor (TNF)-α, and interleukin (IL)-2. A representative flow cytometry plot showing IFN-γ and TNF-α expression is shown. (**F**) NSG mice were subcutaneously injected with the AsPC1 pancreatic cancer cell line. On day 30, when tumor volume reached 300-400mm^3^, mesothelin-directed CAR T-cells were intravenously administered. (**G**) Tumor growth was monitored in AsPC1 tumor-bearing mice. (**H**) NSG mice were intravenously injected with 1 × 10^6^ NALM-6-CBG cells, followed by infusion of 3 × 10^5^ CD19 CAR T-cells (pBBz, negative control), *AAVS1* KO PSMA CAR T-cells, *PRDM1* KO PSMA CAR T-cells, *NR4A3* KO CAR T-cells or *PRDM1/NR4A3* dual KO CAR T-cells at 7-days post-tumor injection (*n* = 7 to 8 per group). Representative bioluminescent images are shown. (**I to L**) Immunophenotyping of CD19 CAR T-cells isolated from NALM-6 engrafted mice at day 24 post-tumor injection. (**I**) The CAR T-cell count in the peripheral blood is shown. (**J**) Frequencies of CD62L-positive CAR T-cells, (**K**) PD-1 and LAG-3 double-positive CAR T-cells and (**L**) PD-1 and TIM-3 double-positive T-cells are shown. Data are shown as scatter points, where the mean and S.E.M. bars are included. . **P* < 0.05, ***P* < 0.01, ****P* < 0.001, n.s., not significant. Data were analyzed using a Kruskal-Wallis test with a post-hoc Dunn’s multiple comparisons test..


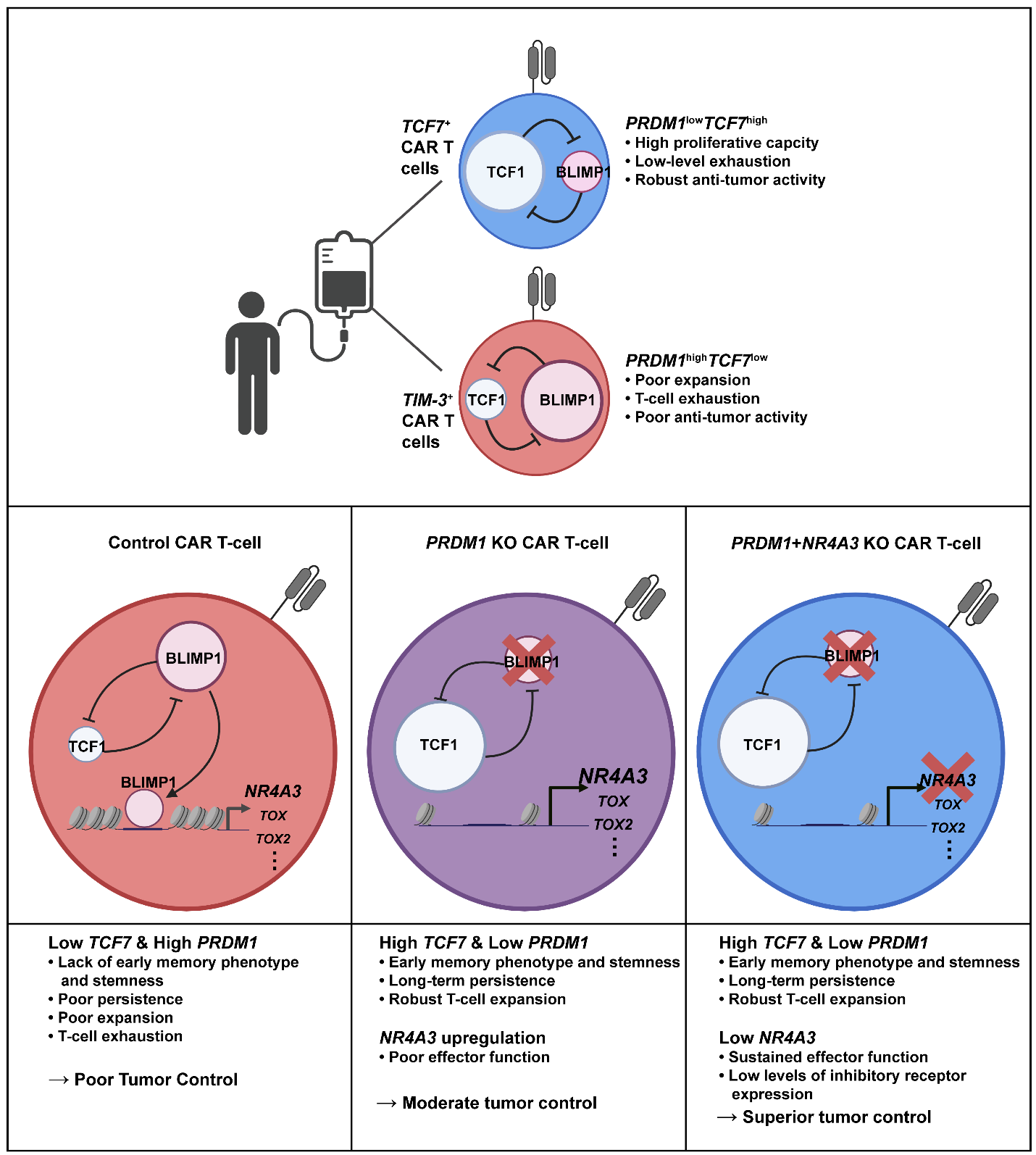


**Figure S8. Graphical summary of how *PRDM1*/*NR4A3* dual ablation enhances CAR T-cell-mediated antitumor efficacy.**

**Data file S1. Raw, individual-level data.**
